# Unique Properties of the Alpha-Helical DNA-Binding Protein KfrA Encoded by the IncU Incompatibility Group Plasmid RA3 and Its Host-Dependent Role in Plasmid Maintenance

**DOI:** 10.1128/AEM.01771-20

**Published:** 2021-01-04

**Authors:** Ewa Lewicka, Monika Mitura, Kamil Steczkiewicz, Justyna Kieracinska, Kamila Skrzynska, Malgorzata Adamczyk, Grazyna Jagura-Burdzy

**Affiliations:** a Institute of Biochemistry and Biophysics, Department of Microbial Biochemistry, PAS, Warsaw, Poland; b Institute of Biochemistry and Biophysics, Department of Bioinformatics, PAS, Warsaw, Poland; c Warsaw University of Technology, Faculty of Chemistry, Chair of Drug and Cosmetics Biotechnology, Warsaw, Poland; University of Tokyo

**Keywords:** alpha-helical plasmidic protein, broad-host-range RA3 plasmid, stability functions, IncU group

## Abstract

KfrA, encoded on the broad-host-range RA3 plasmid, is an alpha-helical DNA-binding protein that acts as a transcriptional autoregulator. The KfrA_RA3_ operator site overlaps the *kfrA* promoter and is composed of five 9-bp direct repeats (DRs). Here, the biological properties of KfrA were studied using both *in vivo* and *in vitro* approaches. Localization of the DNA-binding helix-turn-helix motif (HTH) was mapped to the N29-R52 region by protein structure modeling and confirmed by alanine scanning. KfrA repressor ability depended on the number and orientation of DRs in the operator, as well as the ability of the protein to oligomerize. The long alpha-helical tail from residues 54 to 355 was shown to be involved in self-interactions, whereas the region from residue 54 to 177 was involved in heterodimerization with KfrC, another RA3-encoded alpha-helical protein. KfrA also interacted with the segrosome proteins IncC (ParA) and KorB (ParB), representatives of the class Ia active partition systems. Deletion of the *kfr* genes from the RA3 stability module decreased the plasmid retention in diverse hosts in a species-dependent manner. The specific interactions of KfrA with DNA are essential not only for the transcriptional regulatory function but also for the accessory role of KfrA in stable plasmid maintenance.

**IMPORTANCE** Alpha-helical coiled-coil KfrA-type proteins are encoded by various broad-host-range low-copy-number conjugative plasmids. The DNA-binding protein KfrA encoded on the RA3 plasmid, a member of the IncU incompatibility group, oligomerizes, forms a complex with another plasmid-encoded, alpha-helical protein, KfrC, and interacts with the segrosome proteins IncC and KorB. The unique mode of KfrA dimer binding to the repetitive operator is required for a KfrA role in the stable maintenance of RA3 plasmid in distinct hosts.

## Abstract (precis)

Alpha-helical coiled-coil KfrA-type proteins are encoded by various broad-host-range low-copy-number conjugative plasmids. The DNA-binding protein KfrA encoded on the RA3 plasmid, a member of the IncU incompatibility group, oligomerizes, forms a complex with another plasmid-encoded, alpha-helical protein, KfrC, and interacts with the segrosome proteins IncC and KorB. The unique mode of KfrA dimer binding to the repetitive operator is required for a KfrA role in the stable maintenance of RA3 plasmid in distinct hosts.

## INTRODUCTION

Broad-host-range (BHR) conjugative plasmids act as important vehicles in horizontal gene transfer (HGT) and as such are essential factors in bacterial adaptability and evolution. Their role in spreading advantageous or harmful features in bacterial populations is highly appreciated (1–5). Special attention has been paid to the functions of plasmid backbones in various representatives of the IncP (6–9), IncQ (10, 11), and IncW (12–14) incompatibility groups and their ability to transfer, replicate, and be maintained in phylogenetically distant species.

**FIG 1:**
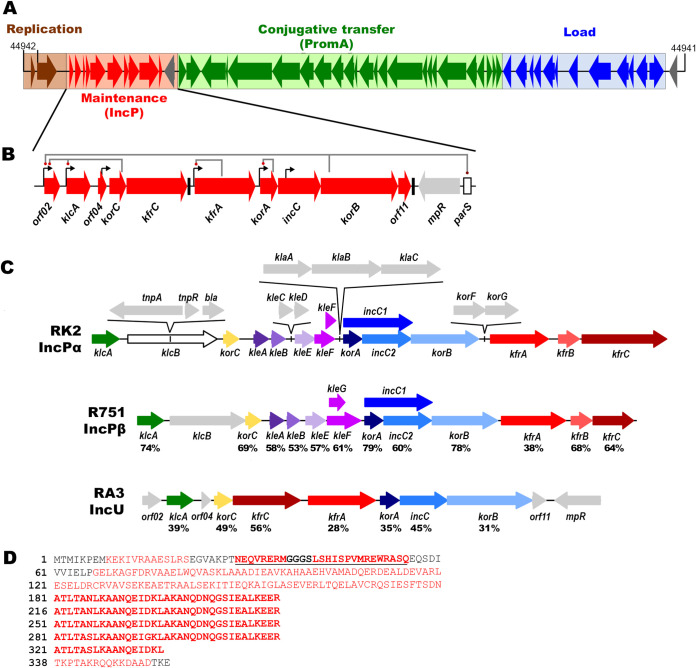
Organization of the stability module of RA3 plasmid (GenBank no. DQ401103.1) from the IncU incompatibility group. KfrA structure predictions. (A) Linear map of RA3 genome (45,909 bp) with functional modules marked with different colors. The closest homologs of ORFs from the stability and the conjugative transfer modules are carried by IncP and PromA plasmids, respectively. The majority of the load cluster corresponds to the class I integron and determines resistance to chloramphenicol, streptomycin, and sulfonamides. Arrows represent ORFs and indicate the direction of transcription. (B) Close-up of the RA3 stability module with its regulatory network. *parS*, a *cis*-acting site in partition, is separated from the partition operon *korA-incC-korB-orf11* by *mpr*, which encodes a putative zinc metalloproteinase homologous to Mpr of pKM101 (GenBank no. AAD17385.1). Black arrows indicate promoters, and black boxes depict Rho-independent transcriptional terminators. Gray lines connect the regulatory genes with the action sites of their products. (C) Comparison of stability modules of representative plasmids from two IncP subgroups and RA3 from the IncU group. Unique ORFs are in gray; homologs found in two or three plasmids are colored similarly. The numbers beneath common ORFs refer to the amino acid sequence identity with RK2 proteins (IncPα). (D) KfrA_RA3_ amino acid sequence with predicted secondary structure (amino acids forming alpha-helical structures are in red). A putative HTH motif is in bold and underlined. Four repetitions of 35 amino acids and one incomplete repetition (17 amino acids) are in bold.

The conjugative, low-copy-number plasmid RA3, isolated from *Aeromonas* spp. (15) is another BHR plasmid of a wide spectrum of hosts belonging to the *Alpha*-, *Beta*-, and *Gammaproteobacteria* (16). Plasmid RA3, the archetype of the IncU group, exhibits a clearly defined modular-mosaic structure with genes seemingly involved in particular plasmid functions, i.e., replication, stability, and conjugative transfer, clustered in operons or blocks of operons that are cooperatively expressed (Fig. 1A). The open reading frames (ORFs) from the stability module and conjugative transfer module have their closest homologs in IncP and PromA plasmids, respectively (8, 9, 16, 17).

In a search for factors determining RA3 species-dependent, long-term retention, we studied the stability module (7.9 kb) of 10 ORFs transcribed in the same direction (Fig. 1B). Recently, we showed that the stability module is organized as a multicistronic operon with at least five differently regulated internal promoters of various strengths (18). Such an organization may provide, on the one hand, tuned expression of the region when required and, on the other, a species-dependent separation of the particular transcriptional units. The main stability mechanism of this module is an active partition system of class 1a (19) encoded in the *korA-incC-korB-orf11* operon with the adjacent centromere-like sequence *parS*. IncC belongs to the ParA family of Walker-type ATPases, while KorB is a DNA-binding protein with an HTH motif and belongs to the ParB family, involved in plasmid and chromosomal DNA segregation to the progeny cells. KorA also contains an HTH DNA-binding motif and autoregulates the expression of the preceding promoter. KorA, IncC, and KorB homologs are present in the IncP plasmids (Fig. 1C) (11, 12), while ORF11, which acts as an accessory protein in the partition process at least in Escherichia coli (20), is unique to IncU plasmids. Another four genes encoding homologs of the IncP proteins, KlcA, KorC, KfrC, and KfrA (Fig. 1C), are localized upstream of the RA3 partition operon and preceded by internal promoters. The gene *klcA*, encoding a putative antirestriction protein (16, 21), is expressed from *orf02p* and *klcAp* (18); both are under the control of the global repressor KorC, and *orf02p* is additionally under the control of KorB (22). *kfrC* and *kfrA* encode alpha-helical proteins that in R751 of the IncPβ group were postulated to support plasmid maintenance (23).

Transcriptional organization of the *kfr* genes in RA3 differs from that in IncP plasmids (Fig. 1C), in which they form a separated tricistronic operon, *kfrA-kfrB-kfrC*, controlled by two global repressors, KorA and KorB (23, 24), by the local KorF regulator (25) and are autoregulated by KfrA (23, 26). In RA3, the *korC-kfrC* genes are expressed from the constitutive *korCp* as well as from two KorC-dependent upstream promoters, *orf02p* and *klcAp* (Fig. 1B). *kfrA* may be expressed from the strongly autoregulated *kfrAp* and additionally from three upstream promoters (18). The *kfrA* gene (KorF regulated) was initially described for the RK2 plasmid of the IncPα incompatibility group (24) and was found to encode a protein of strong autorepressor activity and a very high content of alpha-helices (26). Since then, such DNA-binding, almost entirely alpha-helical proteins have been found encoded not only by IncP and IncU plasmids (16, 23, 26) but also by some BHR conjugative plasmids, classified as members of the PromA and IncW groups (13). Contemporary classification of KfrA proteins is based not on the sequence similarity but on the predicted secondary structure, since even close relatives from RK2 of IncPα and from R751 of IncPβ share only 38% amino acid identity (9). KfrAs of IncP and IncU were postulated to form coiled-coil rod-like structures (16, 23, 26, 27) resembling the SMC (structural maintenance of chromosomes) proteins, although KfrAs lack the ATPase domain characteristic of SMC proteins (28). Additionally, they differ from SMC proteins by their ability to bind DNA in a sequence-specific manner. Under electron microscopy, KfrAs from R751 (IncPβ) and RA3 (IncU) plasmids were found to form short filaments in a buffer with or without plasmid DNA, whereas very long filamentous structures were observed in the presence of plasmid DNA carrying cognate binding sites (M. Adamczyk, E. Lewicka, R. Szatkowska, H. Nieznanska, J. Ludwiczak, M. Jasiński, S. Dunin-Horkawicz, E. Sitkiewicz, B. Świderska, G. Goch, and G. Jagura-Burdzy, submitted for publication). It was shown previously that KfrA of R751 plays an accessory role in the plasmid maintenance in E. coli and Pseudomonas putida (23).

In order to better understand the biological functions of these unique, plasmid-encoded alpha-helical proteins, we analyzed the properties of KfrA from the BHR conjugative RA3 plasmid of IncU group (16, 18). For instance, we assessed KfrA oligomerization both *in vitro* and *in vivo* as well as the specificity of KfrA_RA3_ binding to its operator, O_K_, built of five 9-nt repeats overlapping −35 and −10 motifs of the *kfrA* promoter. The HTH DNA-binding motif in the N-terminal globular part of the KfrA protein was identified. Alanine scanning and thorough analysis of the binding site allowed us to propose the mode of KfrA interactions with the iterative operator. We also found that KfrA forms a complex with KfrC and the segrosome proteins KorB and IncC. The importance of KfrA-O_K_ binding for stable plasmid maintenance was demonstrated, and the role of KfrA and KfrC proteins in the plasmid retention was analyzed in phylogenetically distant bacterial hosts.

## RESULTS

### Protein domains of KfrA.

KfrA of RA3 consists of 345 amino acids with an N-terminal region with a few short alpha-helices followed by a long undisturbed alpha-helical tail (Fig. 1D). This long tail contains four and a half iterations of a 35-amino-acid sequence and a positively charged C-terminal tip. Using Meta-BASIC, we mapped the KfrA_RA3_ N-terminal domain onto multiple transcriptional regulators of known structure, i.e., TrfB (KorA) (PDB code 2N5G), SMIREG2 (PDB code 2Y2Z) and TetR (PDB code 5OJX), with below-threshold confidence scores. The mappings were successfully confirmed by careful manual analysis of the conservation of residues at positions predicted to be important for maintaining the structure and function of the HTH DNA-binding motif (Fig. S1). This analysis pointed out the region between N29 and R52 as a potential helix-turn-helix motif (Fig. 1D) structurally similar to the one present in the transcriptional regulator KorA of RK2 (29).

### Dimerization/polymerization of KfrA.

**FIG 2:**
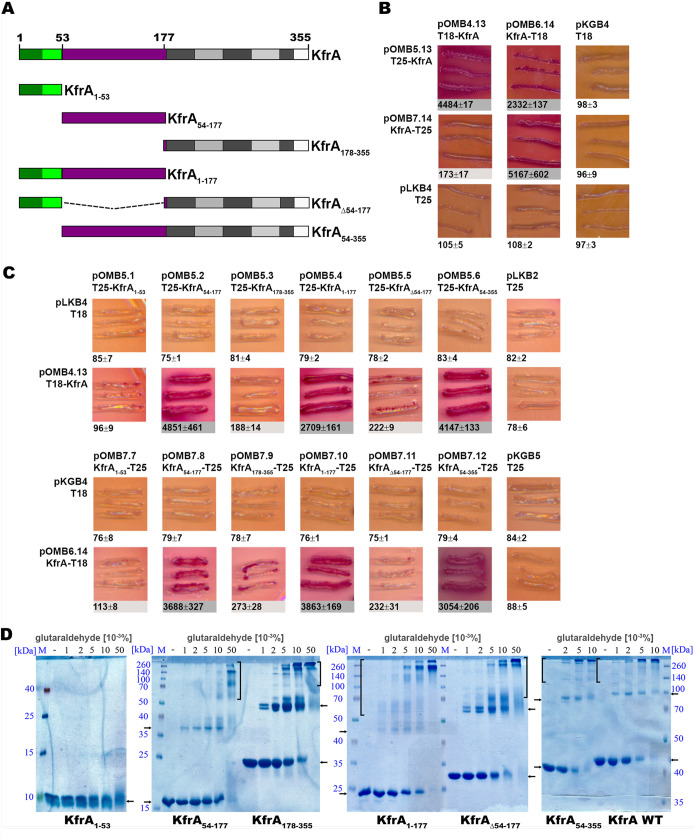
KfrA dimerization *in vivo* and *in vitro*. (A) Graphic presentation of the analyzed *kfrA* deletion mutants. The N-terminal fragment of 53 amino acids is labeled green, with the HTH motif indicated by light green, the middle alpha-helical fragment is purple, and the repetitive motifs in the C terminus are in various shades of gray. Numbers indicate amino acid residues. (B and C) Analysis of KfrA dimerization ability in the BACTH system. Double transformants of E. coli BTH101 *cyaA* with compatible plasmids encoding CyaA fragment T18 or T25 fused to either intact KfrA (B) or various KfrA deletion variants (C) were analyzed on indicator MacConkey plates (pictured here) with maltose as a carbon source and by β-galactosidase assays in liquid cultures. Dark (purple) streaks are indicative of interactions between the two hybrid proteins. Numbers below the images represent β-galactosidase units with SD from at least three experiments. Two shades of gray symbolize strong and weak interactions. Absence of shading indicates no interactions. Double transformants with empty BACTH vectors were used as controls. (D) Cross-linking of purified KfrA variants. His_6_-tagged KfrA and its truncated derivatives (0.1 mg ml^−1^) were incubated with increasing concentrations of glutaraldehyde (0 to 0.05%). The products were separated by SDS-PAGE on polyacrylamide gels of appropriate concentration (18%, 15%, or 12%) and stained with Coomassie brilliant blue. Arrows indicate monomers and putative dimers; brackets encompass higher order complexes. M, protein markers (Spectra multicolor protein ladders [Thermo Fisher Scientific]; low range for KfrA_1–53_ and broad range for the other KfrA variants).

To analyze the KfrA_RA3_ self-association *in vivo*, a bacterial adenylate cyclase-based two-hybrid system (BACTH) was applied (30). WT *kfrA* and its deletion derivatives (Fig. 2A) were cloned into the BACTH vectors to link them translationally with *cyaAT18* and *cyaAT25*, encoding two fragments of the adenylate cyclase (CyaA) catalytic domain. Reconstitution of the CyaA catalytic domain due to interactions between the KfrA molecules led to the activation of catabolic operons, e.g., *mal* and *lac*. This was monitored by growth on MacConkey agar plates with maltose and β-galactosidase assays in liquid cultures of the BTH101 *cyaA* strain transformed with pairs of BACTH vector derivatives (Fig. 2B). Strong interactions between wild-type (WT) KfrA proteins linked to the CyaA fragments from either the N or C terminus were observed on the test plates; however, the highest levels of β-galactosidase activity were detected for pairs of collateral fusion proteins: T18-KfrA/T25-KfrA (4,884 U) and KfrA-T18/KfrA-T25 (5,167 U) (Fig. 2B).

To map the KfrA region involved in dimerization, the *kfrA* gene was split into three fragments encoding (i) KfrA_1–53_, which has a putative DNA-binding domain; (ii) KfrA_54–177_, encompassing the internal alpha-helical fragment; and (iii) KfrA_178–355_, a C-terminal alpha-helical fragment with an iterative 35-amino-acid motif (Fig. 2A). The individual fragments and their combinations were paired with the intact WT *kfrA* in parallel orientation in the BACTH system. N-terminal and C-terminal CyaA-KfrA fusions gave similar results (Fig. 2C). The KfrA_1–53_ fragment did not facilitate significant interactions with the intact KfrA. Strong interactions, comparable to those of WT KfrA-KfrA, were observed for KfrA and the fusion proteins containing KfrA_54–177_, KfrA_1-177_, or KfrA_54–355_. Significant but up to 20-fold weaker interactions were demonstrated between WT KfrA and KfrA_178–355_ or KfrA_Δ54–177_ derivatives. These data suggested that the long alpha-helical tail was capable of dimerization; however, the middle part, KfrA_54–177_, was mainly responsible for KfrA’s ability to self-interact *in vivo*.

The same three *kfrA* fragments and their various combinations were cloned into the pET28 vector. His_6_-tagged polypeptides were overproduced, purified, and tested for the ability to oligomerize in the presence of the cross-linking agent glutaraldehyde (GA) (Fig. 2D). All analyzed polypeptides except KfrA_1–53_ formed dimers and higher-order complexes. Whereas KfrA_178–355_ and KfrA_Δ54–177_ formed mainly dimers, which converted into polymeric complexes only at a high GA concentration, the polypeptides containing residues 54 to 177 (KfrA_54–177_, KfrA_1-177_, and KfrA_54–355_) behaved like WT KfrA and mainly formed the higher-order complexes that hardly entered acrylamide gels. Therefore, *in vitro* analysis confirmed that the region KfrA_54–177_ facilitates *in vitro* polymerization more efficiently than the KfrA_178–355_ region. Additionally, it showed that the C terminus had the potential for dimer formation. This coincided with the *in silico* predictions about coiled-coil regions of KfrA_RA3_ obtained with the use of MultiCoil (31). The fragments encompassing residues 96 to 126 and residues 170 to 339 demonstrated the highest probability of forming coiled-coil structures, trimeric in the former case and only dimeric in the latter case (Fig. S2).

### RA3-encoded partners of KfrA.

Upstream of the monocistronic *kfrA* operon, another presumably alpha-helical protein, KfrC, is encoded in the *korC-kfrC* operon (16). In IncP plasmids, the homologs of KfrA and KfrC are encoded in a tricistronic *kfrA-kfrB-kfrC* operon next to an active partition operon (8, 23), and they have been shown to interact with each other in the yeast two-hybrid system and also postulated to play an accessory role in plasmid active partitioning (23, 26). Hence, the possible interactions not only between KfrA and KfrC of RA3 but also between the Kfr proteins and the segrosome proteins IncC (ParA) and KorB (ParB) were analyzed.

**FIG 3:**
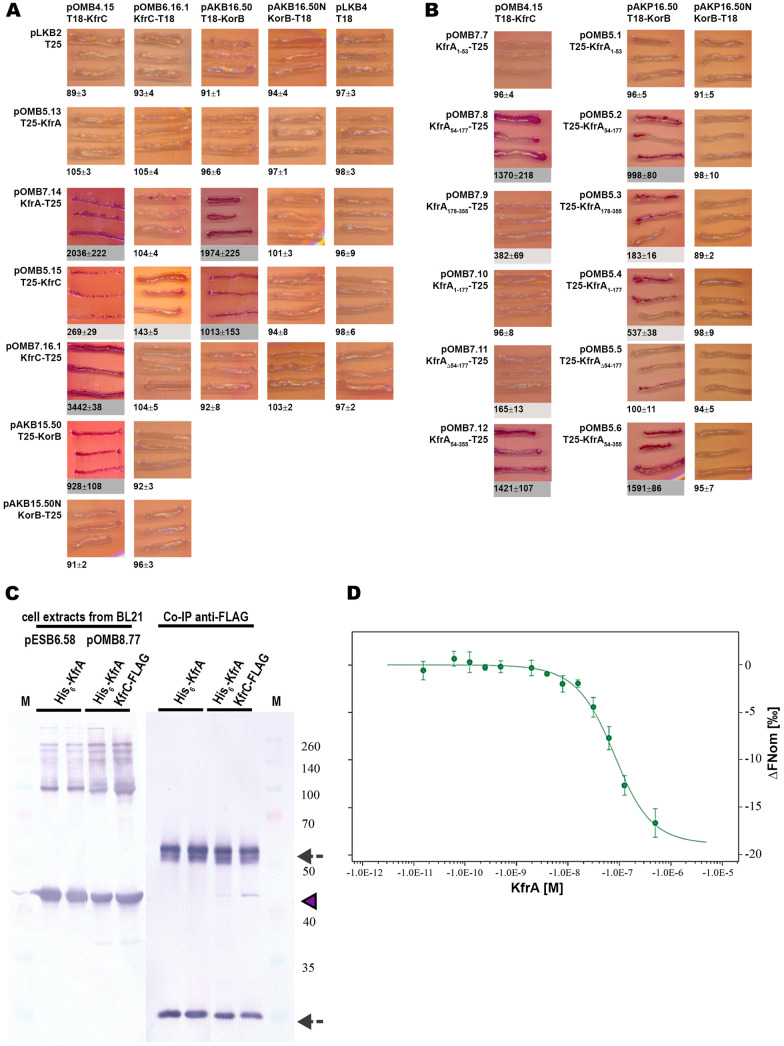
KfrA interactions with its partners, KfrC_RA3_ and KorB_RA3_. (A) Double transformants of E. coli BTH101 *cyaA* with compatible BACTH plasmids encoding CyaA fragments, T18 or T25, fused to KfrA, KfrC, and KorB were analyzed as described in the legend to Fig. 2. (B) Mapping of the KfrA_RA3_ domain involved in the interactions with KfrC and KorB. Experiments were carried out as for Fig. 2. (C) Immunoprecipitation of complexes between KfrA and KfrC. His_6_-KfrA was overproduced in BL21(DE3) either from pESB6.58 (T7p-*his_6_-kfrA*) or together with KfrC-FLAG from pOMB8.77 (T7p- *his_6_-kfrA-kfrC*-FLAG). After immunoprecipitation with anti-FLAG antibodies, proteins were separated by PAGE and screened with anti-His tag antibodies in the Western blot procedure. (Left) Initial cellular extracts (two biological replicates); (right) proteins immunoprecipitated with the use of anti-FLAG antibodies. Arrowhead, His_6_-KfrA (41 kDa); arrows, signals for anti-FLAG antibodies’ heavy and light chains. M, molecular weight markers (kilodaltons). (D) Analysis of the interactions between KfrA and KfrC by microscale thermophoresis (MST). Recombinant His_6_-tagged KfrA_RA3_ and KfrC_RA3_ were overproduced in E. coli B21(DE3) from pAKB2.60 and pESB15.9, respectively, and purified by affinity chromatography. The His_6_ tag was removed from His_6_-KfrA_RA3_, whereas His_6_-KfrC_RA3_ was labeled with RED-Tris-NTA. MST analysis was performed on a Monolith NT.115 instrument at 37°C, with excitation power at 80% and medium with labeled His_6_-KfrC_RA3_ at a constant concentration (50 nM) and a descending gradient of KfrA (from 500 nM to 0.0153 nM). The change in the normalized fluorescence (ΔF_norm_) as a mean value from four independent experiments with standard deviations is shown.

The intact *kfrC* gene was cloned into the BACTH vectors, and complementary pairs of plasmids were introduced into E. coli BTH101 *cyaA*. The strongest KfrC homodimerization was observed for the T18-KfrC/KfrC-T25 pair, suggesting an antiparallel configuration of monomers in the KfrC dimer (Fig. 3A). Strong interactions between KfrA and KfrC of RA3 were detected, confirming that these two alpha-helical proteins formed a complex. Mapping of the KfrA heterodimerization domain led to the suggestion that both alpha-helical fragments KfrA_54–177_ and KfrA_178–355_ are partly involved in the interactions with the KfrC partner (Fig. 3B), although KfrA_54–177_ exhibited much stronger association with KfrC than the C-terminal fragment.

Interactions between KfrA and KfrC were then confirmed *in vitro* by coimmunoprecipitation (Fig. 3C). His_6_-tagged KfrA and KfrC-FLAG were overproduced from the expression vector pOMB8.77 in E. coli BL21(DE3). Placing both ORFs under the same transcriptional signals in one plasmid was expected to secure at least their equimolar transcript dosage. Proteins were cross-linked with the use of formaldehyde and immunoprecipitated with anti-FLAG antibodies. Proteins from the immunoprecipitates (IP) were separated by PAGE and analyzed by Western blotting with anti-His tag antibodies. The extract from BL21(DE3)(pESB6.58 *tacp-his_6_-kfrA*) was treated as a control sample and underwent the same procedure. Detection of His_6_-KfrA only in IP samples where KfrC-FLAG was present verified interactions between KfrA and KfrC observed *in vivo*. The binding affinity of KfrA_RA3_ and KfrC_RA3_ was quantified using microscale thermophoresis (MST), an ultrasensitive method that allowed determination of the complex formation kinetics by detecting variations in a fluorescence signal coming from one of the binding partners (32). To study KfrA-KfrC interactions, we chose the second-generation NT-674 dye for target protein labeling, which binds selectively and noncovalently to the histidine tag and has a minimal impact on the biochemical and physicochemical properties of the protein. KfrA_RA3_ titration of the labeled His_6_-KfrC_RA3_ yielded a *K_d_* of 54 ± 9 nM (Fig. 3D), confirming specific complex formation between KfrA and KfrC *in vitro*.

It was previously shown that KorB_RA3_ formed dimers in the BACTH system mainly when the CyaA fragments was linked to its N terminus, whereas IncC_RA3_ self-associated when its N terminus was free (33). Both proteins, KorB_RA3_ and IncC_RA3_, interacted with each other, and the strongest interactions occurred between the translational fusions of IncC-T25 and T18-KorB (33). Analysis of Kfr proteins interactions with segrosome proteins IncC and KorB revealed that both KfrA and KfrC were capable of interacting efficiently with KorB (Fig. 3A), indicated by 1,974 U and 1,013 U of β-galactosidase activity, respectively. The KfrA domain involved in the interactions with KorB was mapped to the KfrA_54–177_ fragment (Fig. 3B). The same fragment, KfrA_54–177_, facilitated efficient reconstitution of CyaA activity by heterodimerization with KfrC, suggesting that the middle part of KfrA has the ability to interact with various proteins. Weak interactions were also detected between KfrA-T18 and IncC-T25 (136 U of β-galactosidase in comparison to 90 U for control plasmids).

### DNA-binding activity of KfrA_RA3_.

**FIG 4:**
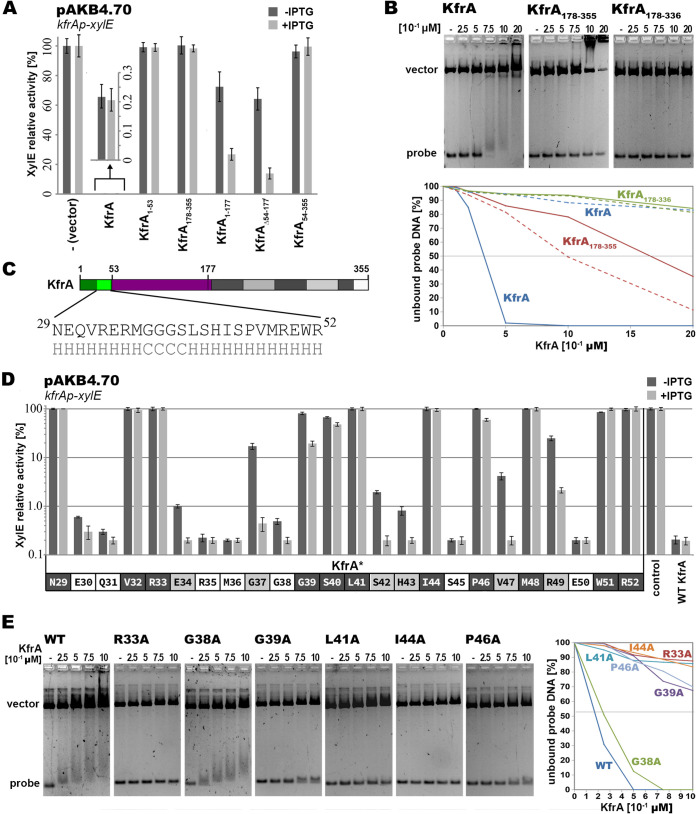
Analysis of HTH motif in KfrA_RA3._ (A) Ability of KfrA deletion variants to repress the *kfrA* promoter. E. coli C600K carrying pAKB4.70 (*kfrAp-xylE*) was transformed with the expression vector pGBT30 or its derivatives encoding WT KfrA and the truncated derivatives. Double transformants were grown without or with 0.5 mM IPTG to induce *tacp* and protein overproduction. XylE activities were assayed in extracts from the exponentially growing cultures and are shown relative to the XylE activity in C600K(pAKB4.70)(pGBT30) cells. Since WT KfrA exhibits strong repression activity (even in the absence of inducer due to the leakiness of *tacp*), the bracketed part of the diagram is enlarged and presented using a different scale. (B) Schematic presentation of KfrA. The color code is as in Fig. 2A. The protein region with the putative HTH motif is enlarged, and the predicted secondary structure (H, helix; C, coil) is shown. (C) The C tip of KfrA_RA3_ is responsible for nonspecific DNA binding activity. His_6_-tagged KfrA and two deletion variants were analyzed by EMSA. Different concentrations (0 to 0.2 μM) of His_6_-tagged proteins were incubated for 30 min at 37°C with 10 nM pESB2.68 DNA (pUC18 carrying *kfrAp*) cleaved into two EcoRI-NdeI fragments: a shorter one with the *kfrAp* sequence (approximately 0.3 kb) and a longer one used as a control (2.4 kb). Nucleoprotein complexes were separated by electrophoresis on 1.7% agarose gels in 1× TBE buffer and visualized by ethidium bromide staining. The graph presents the quantitative analysis of results carried out with ImageJ 1.52N software (71). The percentage of unbound DNA was plotted against the protein concentration applied; continuous lines correspond to the *kfrAp* fragments (probe), and dashed lines correspond to the control fragments (vector) in these three reactions. (D) Alanine scanning of KfrA HTH motif. The putative HTH motif residues were replaced by alanines. Mutagenized *kfrA* alleles were cloned into the expression vector pGBT30 and tested in a two-plasmid regulatory system for the ability to repress *kfrAp-xylE* transcriptional fusion (pAKB4.70). Double transformants of E. coli C600K were grown without or with 0.5 mM IPTG to induce *tacp* and protein overproduction. XylE activities were assayed in extracts from exponentially growing cultures and shown on diagram relative to the activity of C600K(pAKB4.70)(pGBT30) using a logarithmic scale. KfrA* represents KfrA derivatives with amino acid substitutions. Amino acids shaded in dark gray indicate those in which Ala substitutions led to significant impairment, Ala substitutions for those shaded in light gray led to partial impairment, and strains with Ala substitutions for unshaded amino acids retained WT repression ability. The last two sections on the right correspond to the XylE activities in the control strains C600K(pAKB4.70)(pGBT30) and C600K(pAKB4.70)(pESB5.58 *tacp-kfrA*) grown with and without IPTG. (E) DNA binding activity of KfrA single-substitution variants *in vitro*. Selected His_6_-tagged KfrA variants were used in the EMSA with NdeI-EcoRI-cut pESB2.68 (pUC18 carrying *kfrAp*) as described in the legend to panel C. The graph illustrates the quantitative analysis of the results obtained with the use of ImageJ 1.52N software (71). The percentage of unbound DNA (probe) was plotted against the protein concentration applied.

It has been predicted that KfrA_RA3_ may autoregulate monocistronic *kfrA* operon expression, as has been shown for IncP plasmids (16, 23, 26). The *kfrA* promoter region was inserted into the promoter-probe vector pPT01 upstream of the *xylE* cassette (pAKB4.70) and demonstrated the high transcriptional activity (2 U of XylE) in E. coli C600K transformants. When the *kfrA* gene was cloned under the control of *tacp* (pESB5.58) in the multicopy expression vector pGBT30 (25) and introduced into E. coli C600K(pAKB4.70), it repressed *xylE* expression 500-fold even without IPTG (isopropyl-β-d-thiogalactopyranoside) induction (Fig. 4A).

Various *kfrA* deletion alleles (Fig. 2A) were cloned into pGBT30 and tested for the ability to repress the *kfrAp-xylE* transcriptional fusion in the two-plasmid regulatory system (Fig. 4A). Removal of the N-terminal 53 residues with the putative HTH motif abolished the repressor activity of the truncated KfrA_54–355_. The N-terminal KfrA_1–53_ fragment was incapable of repression on its own; however, when linked to the alpha-helical regions either between residues 54 and 177 (KfrA_1-177_) or between residues 178 and 355 (KfrA_Δ54–177_), it partially repressed *kfrAp* (up to 10-fold in the presence of the protein excess compared to the 500-fold repression by WT KfrA). Since both these KfrA fragments seem to be involved in self-association (Fig. 2), it implicates that KfrA acts as a dimeric repressor and that the domain responsible for DNA binding is indeed located within the first 53 amino acids. The purified His_6_-tagged KfrA fragments were tested for DNA binding in the electrophoretic mobility shift assay (EMSA) with digested pUC18 carrying *kfrAp* (pESB2.68). Under these conditions, the intact His_6_-tagged KfrA was capable of specific DNA binding (Fig. 4B), with a *K*_apparent_ (*K*_app_) of approximately 0.32 μM. (*K*_app_ is determined experimentally in electrophoretic mobility shift assays [EMSA] as the concentration of DNA-binding protein at which 50% of specific DNA is bound.) For different preparations of WT KfrA, *K*_app_ varied in the range 0.18 to 0.32 μM. Among other tested fragments, only KfrA_178–355_ demonstrated DNA binding activity at the applied range of protein concentrations; however, it shifted both fragments, a smaller fragment with *kfrAp* (*K*_app_, 1.7 μM) and a bigger control one with a *K*_app_ of 1 μM. Deletion of the C-terminal 19 amino acids in KfrA_178-336_ abolished this activity (Fig. 4B), suggesting that the positively charged C terminus of KfrA (Fig. 1D) was responsible for the nonspecific DNA binding.

Further studies were performed on the *kfrA* point mutants with alanine substitutions of the subsequent residues in the putative HTH motif (Fig. 4C). All mutated *kfrA* alleles were cloned into the expression vector pGBT30 under the control of *tacp* and tested *in vivo* for the repressor ability for *kfrAp* in the two-plasmid regulatory system (Fig. 4D). This analysis validated the proper *in silico* identification of the DNA binding motif, since 17 of 24 KfrA variants with a single Ala substitution in this region were impaired to some extent in *kfrAp* repression. For 10 KfrA variants, i.e., those with the mutations N29, V32, and R33 in the binding helix and S40, L41, I44, P46, M48, W51, and R52 in the recognition helix of the HTH motif, the repression was almost completely abolished (even at the high KfrA concentration). Among three glycine residues in the turn region, G39 seems to play the most important role in the proper positioning of the helices. To confirm the *in vivo* results, representative *kfrA* alleles were cloned into pET28mod, and the His_6_-tagged versions of KfrA variants were purified and used in EMSA with digested plasmid DNA (pESB2.68) containing the *kfrAp* region (Fig. 4E). WT KfrA and KfrAG38A demonstrated *K*_app_ values of 0.18 μM and 0.26 μM, respectively. Lack of DNA binding in the tested range of protein concentrations correlated with the loss of repressor activity by KfrAR33A, KfrAL41A, and KfrAI44A, whereas partial impairment of the repressor activity *in vivo* of KfrAG39A and KfrAP46A (Fig. 4D) was observed as significantly decreased DNA binding affinity *in vitro* (Fig. 4E).

### KfrA binding site requirements.

**FIG 5:**
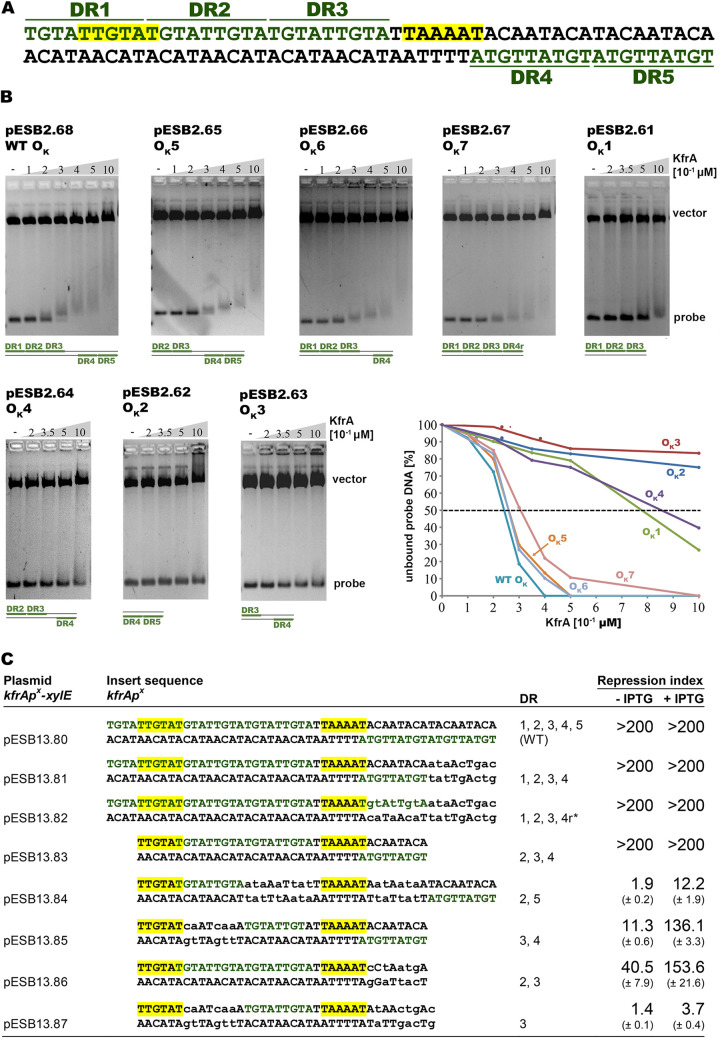
Analysis of KfrA binding site (O_K_). (A) DNA sequence of minimal *kfrAp*. The −35 and −10 motifs of the promoter are marked in yellow. Five 9-nt repeats (DR) surrounding the −10 motif (three direct repeats in one direction and two in the opposite orientation) are numbered and indicated with lines. (B) Ability of KfrA to bind to a DR(s) *in vitro*. Oligonucleotides corresponding to various combinations of DRs (in number and orientation) were cloned into pUC18; purified plasmid DNA was digested with NdeI and EcoRI and used in EMSAs with different concentrations (0 to 0.1 μM) of purified His_6_-KfrA. DNA-protein complexes were separated by electrophoresis on 1.7% agarose gels in 1× TBE buffer and visualized by ethidium bromide staining. Schemes of DR combinations cloned in pUC18 are shown below the gels. The graph illustrates the quantitative analysis of results carried out with ImageJ 1.52N software (71). The percentage of unbound DNA (probe) was plotted against the concentrations of KfrA applied. (C) Analysis of *kfrAp* variants with different combinations of DRs *in vivo*. Double-stranded oligonucleotides encompassing the −35 and −10 motifs of *kfrAp* and DRs in various numbers and orientations were cloned into the promoter probe vector pPT01. Sequences of the oligonucleotide inserts are shown, with promoter motifs highlighted in yellow and DRs in green. Lowercase letters indicate introduced substitutions in comparison with the WT sequence. Repeats listed in the DR column were retained intact. pPT01 derivatives were introduced into E. coli C600K and analyzed in the two-plasmid regulatory system with empty expression vector pGBT30(*tacp*) or pESB5.58(*tacp-kfrA*) in *trans*. Cultures of double transformants were grown without or with 0.5 mM IPTG to induce protein overproduction and XylE activity was measured in extracts of exponential-phase cultures. The KfrA repression index and the standard deviation (SD) were calculated for each promoter region as a ratio of XylE activity in the presence of pGBT30 to the XylE activity in the presence of pESB5.58 grown under the same conditions. The assays were repeated at least 3 times.

A short intergenic fragment of 130 bp between *kfrC* and *kfrA* contains a Rho-independent transcriptional terminator (18) followed by the *kfrA* promoter motifs (16). The −35 motif, TTGTAT, is embedded in the three DRs of 9 nt (TGTATTGTA), whereas the −10 motif, TAAAAT, separates these three adjacent repeats from the remaining two oppositely oriented copies (Fig. 5A). It was postulated (16) that these repeats composed the unusually structured KfrA binding site (O_K_), which facilitated extremely efficient KfrA repression of *kfrAp*. Beyond this location, the 9-nt motif TGTATTGTA was not found in the RA3 genome.

To define minimal requirements for KfrA binding to DNA *in vitro* as well as its ability to repress the promoter *in vivo*, this putative KfrA operator was further dissected. Oligonucleotides corresponding to the various numbers of DRs and their different arrangements were synthesized and cloned into pUC18; plasmid DNAs were then purified and digested, and KfrA binding was analyzed *in vitro* by EMSA (Fig. 5B). The data obtained led to the conclusion that DNA fragments with five repeats (intact WT O_K_) were bound by KfrA with the highest affinity (*K*_app_, 0.24 μM). Deletion of one 9-nt repeat at either end of the set (O_K_5 and O_K_6) did not significantly change KfrA binding (*K*_app_, 0.26 μM), demonstrating that four repeats were sufficient for KfrA association. Both O_K_5 and O_K_6 contained at least two repeats (DR3 and DR4) in an inverted orientation (Fig. 5A). To establish whether such a palindromic orientation was vital for KfrA dimer binding, the four repeats were compiled in the same orientation (O_K_7) and shown to be bound by KfrA with an only slightly decreased affinity (*K*_app_, 0.31 μM) in comparison to O_K_6. DNA fragments with three DRs (O_K_1 and O_K_4) were bound by KfrA with at least 3-fold-lower affinity than WT O_K_ (Fig. 5B). Decreasing the number of repeats to two (O_K_2 and O_K_3) abolished KfrA binding under the conditions used.

An atypical structure of O_K_ in RA3 prompted *in vivo* studies on the role of the DRs and their orientation in the repressor function of KfrA. Two annealed 52-nt complementary oligonucleotides were cloned into the promoter-probe vector pPT01 upstream of the *xylE* cassette (pESB13.80) and confirmed to carry an intact *kfrAp*. The XylE activity assayed in extracts of exponentially growing E. coli C600K(pESB13.80) was approximately 2 U, similar to the activity of *kfrAp* from a 130-bp intergenic fragment previously cloned in pAKB4.70. Seven additional variants of the *kfrA* promoter region with various numbers of DRs were synthesized and cloned into pPT01 (Fig. 5C). WT and mutant *kfrAp*s were analyzed in the two-plasmid regulatory system with pGBT30 (expression vector) or pESB5.58 (*tacp-kfrA*) in *trans*. Double transformants of E. coli C600K were grown to the exponential phase, in the presence of selective antibiotics and with or without 0.5 mM IPTG inducer. Strong repression of WT *kfrAp* (repression index [RI] of more than 200) was observed even at a low level of KfrA production (without *tacp* induction), as shown in Fig. 5C. *kfrAp* variants with four DRs in various orientations to each other (1/2/3/4 and 1/2/3/4r) or three DRs (1/2/3) demonstrated a sensitivity to the presence of KfrA *in vivo* similar to that of WT *kfrAp* with five DRs. Decreasing the number of repeats to two, in either an inverted (3/4) or direct (2/3) orientation, significantly diminished the ability of KfrA to repress *kfrAp*, especially under low KfrA abundance (RI of 10 to 40 without KfrA overproduction). The construct with two inverted repeats separated by 25 bp (2/5) or a single 9 bp unit (pESB13.87) was hardly affected by KfrA presence (Fig. 5C) in terms of *kfrAp* activity compared to the other tested promoter regions. Together, these data confirmed the autoregulatory function of KfrA and furthermore suggested that at least two 9-nt motifs close to each other were required and sufficient for KfrA binding and transcriptional regulation of *kfrAp in vivo*.

### KfrA-DNA binding is required for stable plasmid maintenance in E. coli.

**FIG 6:**
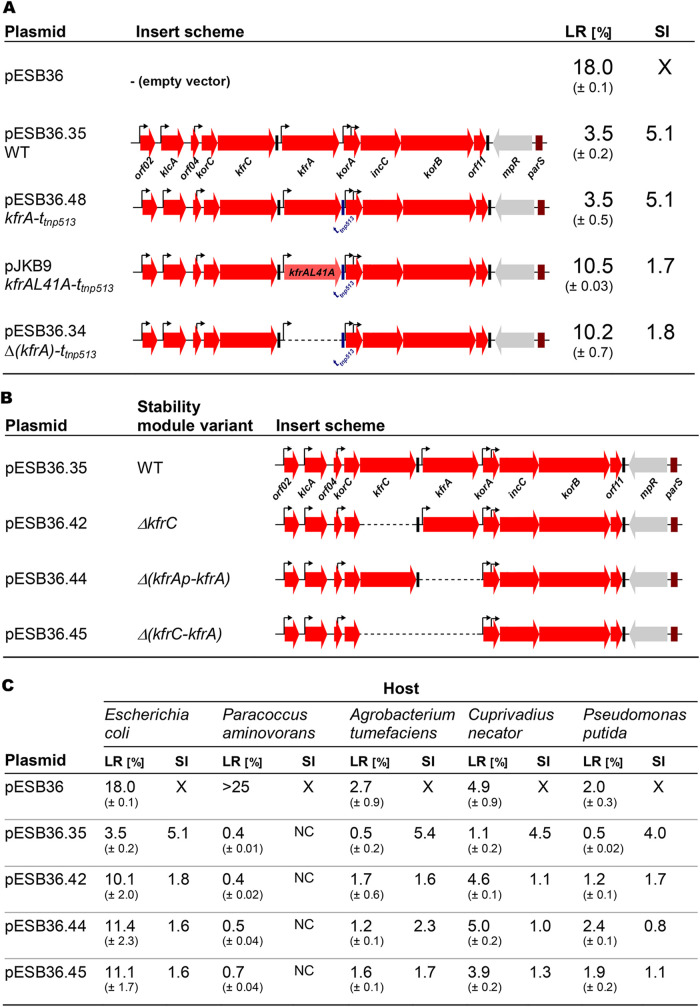
Significance of the *kfrC-kfrA* region in the stability potential of the RA3 stability module in various hosts. (A) KfrA binding to its operator is vital for plasmid stability in E. coli. The stability module of RA3 and its modified variants were cloned into the low-copy-number, highly unstable vector pESB36. Inserts are schematically shown, with thick arrows representing genes, thin black arrows indicating promoters, and black boxes marking positions of native Rho-independent transcriptional terminators. Blue bars indicate a strong Rho-independent transcriptional terminator introduced into the stability cassette to protect the partition operon from RNA polymerase read-through due to planned genetic manipulations. The extent of *kfrA* deletion is shown as a dashed line. E. coli EC1250 transformants of pESB36 and its derivatives were grown without selection for 60 generations, and plasmid loss rates per generation (LR) were established. The stability indexes (SI) were calculated by dividing LR of an analyzed plasmid by LR of the empty vector. (B) Schematic presentation of *kfrC-kfrA* deletion derivatives of the RA3 stability module cloned into the unstable vector pESB36 (descriptions are as for panel A). (C) Segregation of pESB36 and its *kfrC-kfrA* deletion derivatives in five hosts. Test plasmids from panel B were introduced into various RA3 hosts from the *Alphaproteobacteria* (A. tumefaciens), *Betaproteobacteria* (*P. aminovorans* and C. necator), and *Gammaproteobacteria* (E. coli and P. putida). At least three biological replicates of strains harboring analyzed plasmids were grown without selection for 60 generations, and plasmid loss rate per generation (LR) and stability index (SI) were established. X, irrelevant; NC, not calculable.

A previous study (18) indicated that the low-copy-number, very unstable miniRK2 replicon (pESB36), with a loss rate (LR) of 18% per generation, was stabilized in E. coli by the presence of the 7.7-kb RA3 stability module (pESB36.35) including the *kfrC* and *kfrA* genes (Fig. 6A). It has also been shown that the *kfrA* operon of RA3 was vital for heterologous plasmid maintenance in E. coli and significantly important in other hosts (18). Due to the detected read-through along the stability module, previous work was done on the plasmid with the deletion of *kfrAp-kfrA*. Here, to analyze the roles of the intergenic region encompassing *kfrAp* with O_K_ and KfrA on its own in plasmid stability, the new constructs were used. It was decided to exchange WT *kfrA* for the mutant allele encoding KfrAL41A, which was unable to bind to DNA and repress *kfrAp*. To avoid RNA polymerase (RNAP) read-through from the strong unregulated *kfrAp* into the downstream partition operon, first the extremely efficient Rho-independent terminator t*_tnp513_* (34) was inserted downstream of the *kfrA* gene in pESB36.35 to obtain pESB36.48 (Fig. 6A). Next, the *kfrA* ORF was either removed (pESB36.34) or replaced by *kfrA*L41A (pJKB9). Retention of all plasmids was analyzed in E. coli EC1250 strain for up to 60 generations of growth without selection. Both derivatives, pESB36.34 and pJKB9, demonstrated the same high rate of the plasmid loss (10% per generation), confirming that the absence of KfrA or even a defect in its DNA binding activity is deleterious for plasmid maintenance in E. coli.

### Role of KfrA and KfrC in plasmid maintenance in various hosts.

The bacterial two-hybrid analysis clearly demonstrated the interactions between KfrA and KfrC at least in E. coli (Fig. 3). Different deletion derivatives of pESB36.35 were constructed in the region encoding both Kfr proteins (Fig. 6B) and tested for stability in E. coli and four other RA3 hosts: P. putida (another member of the *Gammaproteobacteria*), Paracoccus aminovorans and Agrobacterium tumefaciens (members of the *Alphaproteobacteria*), and Cupriavidus necator (a member of the *Betaproteobacteria*) (Fig. 6C). It was shown previously (18) that the pESB36 vector was unstable in the tested species but to various extents. The highest loss rate was observed in *P. aminovorans* (estimated LR more than 25% per generation), and the lowest LR of 2% per generation was observed in P. putida. The presence of the RA3 stability module in pESB36.35 significantly improved the retention of the plasmid, with a stability index of 4 to 5 in all species but *P. aminovorans*, in which the SI was estimated to be more than 60. In E. coli, the deletion of either the *kfrA* ORF or the *kfrC* ORF (or both) led to plasmid instability, with a loss rate of 10 to 11% per generation (Fig. 6C). In P. putida, both proteins were also important for test plasmid stability. Interestingly, the deletion of the *kfrA* operon led to a slightly higher loss rate than the deletion of *kfrC* or both *kfrC* and *kfrA*, suggesting that KfrC protein not complexed with KfrA might additionally negatively affect this host. Also in C. necator, the deletion of either of the *kfr* genes led to the destabilization of the plasmids and a 4- to 5-fold-higher plasmid loss rate per generation than observed for pESB36.35. Two members of the *Alphaproteobacteria* differed in the requirements for the Kfr proteins to stabilize the test plasmid. Whereas the deletion of *kfr* genes hardly affected plasmid stability in *P. aminovorans*, both proteins were required for the stable maintenance of the test plasmid in A. tumefaciens. Overall, this analysis confirmed the participation of the KfrA-KfrC complex in the stability of the low-copy-number plasmid that relied on the class Ia active partition system. It also showed that the significance of Kfr proteins varied in a species-dependent way.

## DISCUSSION

Alpha-helical, filament-forming proteins play a wide spectrum of roles in the prokaryotic cells (reviewed in references 35 and 36). They function as structural and motor proteins in cell shaping (e.g., MreB [37] and crescentin [38]), cell division (e.g., the FtsZ-FtsA complex [39, 40]), chromosome segregation (e.g., the SMC/MukB complexes [41, 42]), and organization of intracellular components (e.g., MamK [43]). Remarkably, there are also plasmid-encoded filamentous alpha-helical proteins. Actin-like ATPases (ParM proteins [44, 45]) and tubulin-like GTPases (TubZ proteins [46, 47]) build dynamic structures that facilitate active segregation of the plasmid molecules to the progeny cells by pushing (class II active partition system) or tramming (class III) (19, 48).

Another plasmid-encoded alpha-helical myosin/kinesin-like protein was originally found encoded on IncP plasmids (23, 26) and designated KfrA. At present, KfrA proteins, defined as alpha-helical proteins with N-terminal DNA-binding domains, have been annotated for numerous broad-host-range plasmids in the IncP, IncU, IncW, and PromA groups (13, 16, 17, 23, 26, 49–51). The specific role of KfrAs in the biology of these low-copy-number plasmids remains enigmatic, although at least the representative of the IncP group (23), KfrA_R751_, seems to participate in the plasmid stable maintenance. Plasmids encoding KfrA proteins rely in their segregation on the most commonly occurring active partition systems of class Ia (19), based on the dynamic gradient of Walker-type ATPases (ParA, SopA, and IncC proteins) that separates and relocates segrosomes, i.e., nucleoprotein complexes between specific DNA-binding proteins (ParB, SopB, and KorB proteins) and cognate plasmid DNAs (52, 53).

In this study, it was confirmed that the alpha-helical protein KfrA of plasmid RA3 (IncU) bound to the single locus O_K_ in the plasmid genome. KfrA_RA3_ acts as the only and a very potent transcriptional regulator of the monocistronic *kfrA* operon, whereas its IncP counterparts, encoded by tricistronic *kfrA-kfrB-kfrC* operons, are supported in their autoregulatory repression by two global regulators, KorA and KorB (23, 26). The KfrA_RA3_ binding site O_K_ is unique because of its unusual repetitive character. It is built of five 9-nt DRs (2 of them oppositely oriented) that encompass *kfrAp* motifs. It has been shown that at least two DRs are required for repression of *kfrAp* but are not sufficient for switching off the promoter. The presence of 4 repeats, regardless of their orientation, is required for full repression.

**FIG 7:**
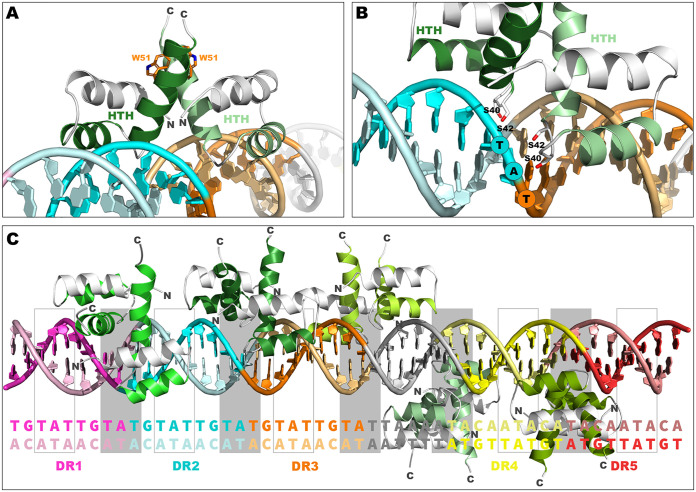
Positioning of KfrA HTH domains at *kfrAp*. (A) 3D model of two KfrA HTH domains binding to a single DNA major groove. (B) More detailed view of KfrA-DNA interaction. Serine residues contributing to DNA binding are rendered as sticks. (C) Model of the promoter region with docked KfrA dimers. TAT sequences at DR interfaces are highlighted in gray rectangles and occupied by KfrA dimers, while others are outlined only for clarity. The DNA sequence of the promoter is given below the corresponding base pairs in the model.

A comparative three-dimensional (3D) model of the KfrA HTH domain, based on the structure of TrfB (KorA) used as a template (29), demonstrates that it exposes only a few polar amino acids to the DNA double helix, namely, S40, S42, S45, and R49 in a recognition helix and R33 in a binding helix. Interestingly, the recognition helix harbors totally conserved tryptophan (W51), which is unlikely to interact with the DNA but might be involved in dimerization. The role of this residue becomes apparent in a KfrA dimer model assembled on the structure of the plasmid segregation protein TubR, which dimerizes through the recognition helix (PDB code 3M8E) (54). Such mutual orientation of KfrA proteins would bring the tryptophan residues closer, which might allow their stacking (Fig. 7A) and, in consequence, would additionally stabilize the dimer. In such a case, the recognition helices of the KfrA dimer, as in the case of TubR, would fit into a single major groove, unlike other canonical HTH proteins, in which the recognition helices insert singly into the successive major grooves.

Alanine scanning confirmed the essential role of this tryptophan residue in the KfrA repressor function (Fig. 4D). In such a dimer, S40 would be exposed to the DNA phosphate backbone for sequence-independent DNA binding, while S42 would scan the major groove with its hydroxyl group (Fig. 7B). Presented experiments confirmed the important role of S40 in DNA binding; however, S42 was not exclusively responsible for DNA recognition (Fig. 4D). Its substitution by alanine only mildly disrupted proper KfrAS42A-O_K_ interactions, indicating the involvement of other, unidentified factors stabilizing KfrA at its target site. Solving the crystal structure of KfrA dimers, or at least of N-terminal parts of proteins with shortened alpha-helical tails, complexed with the operator, should verify the proposed model.

Based on the derived repression indexes for modified KfrA binding sites, we hypothesized that the KfrA dimer might bind to interfaces between DRs (TAT sequence) (Fig. 7B). It is noteworthy that there are multiple TAT sequences within the operator region (Fig. 7C); the saturation of possible KfrA binding sites in *kfrAp* may determine the strength of KfrA repression, or restructuring of the complex may be correlated with possibly different roles the protein plays.

Here, we showed that the specific DNA binding activity of KfrA_RA3_ to its unique operator was required for the stable maintenance of the test plasmid in different hosts (Fig. 6A and C). It has been suggested that KfrA of IncP may form a scaffold for plasmid molecules to be transported to the dedicated cellular positions by the active partition system of class Ia before cell divisions (23, 26). We report here evidence for direct interactions between KfrA_RA3_ and plasmid active partitioning complex, strong interactions with the segrosome-forming protein KorB, and weak interactions with IncC, the partner of KorB in the segregation process.

Additionally, it was shown that KfrA formed a complex with another alpha-helical plasmid-encoded protein, KfrC (Fig. 3B to D), and both proteins participated in stable plasmid maintenance, as shown in our test plasmid system (Fig. 6C). The domain of heterodimerization with two partners, KfrC and KorB, was mapped to the middle part of KfrA, i.e., KfrA_54–177_ (Fig. 3B), which implicated the competitive binding between two KfrA partners. The role of the KfrA-KfrC complex varied from vital to redundant in different bacterial strains. The redundancy observed in *P. aminovorans* suggests that the partition mechanism is self-contained or it uses accessory proteins that are chromosomally encoded or that possibly other parts of the stability cassette (antirestriction protein KlcA or Orf02 of unknown function) play dominant roles in the plasmid stable maintenance in this species.

**FIG 8:**
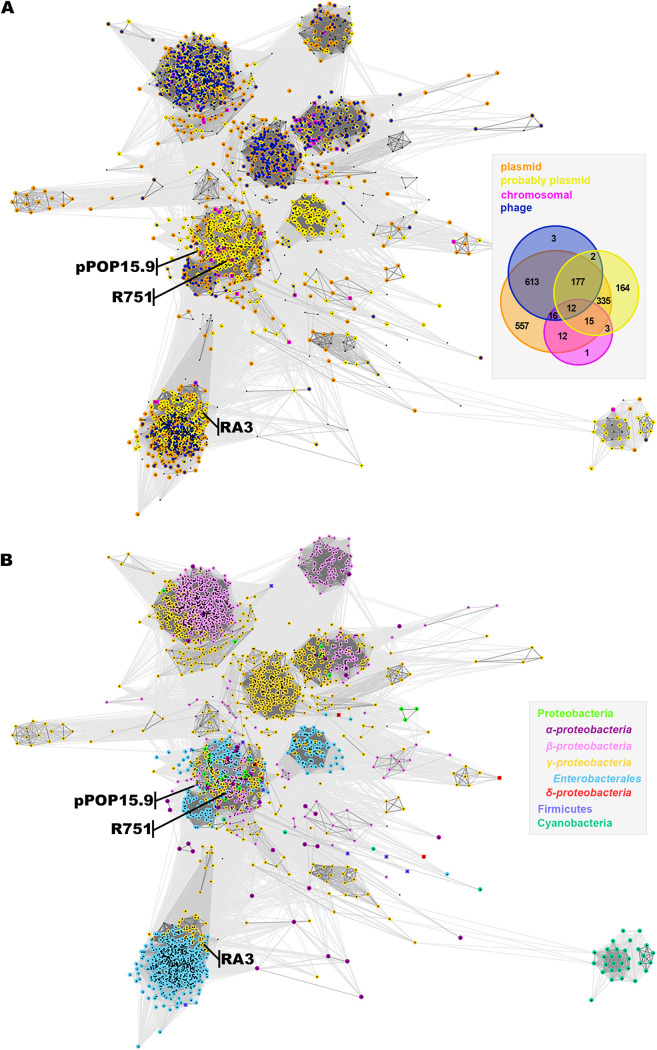
Distribution of KfrA proteins with homologous DNA binding domain. (A) Clustering of 1,909 KfrA_RA3_ homologs according to CLANS (85). The color code corresponds to the putative origin of *kfrA* genes based on their genomic neighborhood (Venn diagram). (B) Phylogenetic analysis of *kfrA*-carrying genomes. Mobile elements isolated from unknown hosts were generally classified as belonging to the phylum *Proteobacteria*, *Firmicutes*, or *Cyanobacteria*. Known hosts facilitated classification to the classes *Alpha*-, *Beta*-, *Gamma*-, and Deltaproteobacteria and more specifically to the order *Enterobacterales* in the *Gammaproteobacteria*.

Sequence searches using the N domain of KfrA of RA3 as a query revealed more than 2,600 homologs, all containing alpha-helical tails of various lengths. Since the majority of the annotated sequences originated from the fragmentary assembled genomes, in order to assess whether a particular sequence comes from a plasmid or a phage or is chromosomal, the ORFs in the immediate vicinity (three up and three down) of the *kfrA*-like genes were analyzed. A total of 1,909 *kfrA* genes were found to localize in the neighborhood of at least one plasmid- or phage-related ORF (Fig. 8A). The majority of *kfrA* genes were surrounded by genes encoding protein domains characteristic of plasmids (1,086 genes) or both plasmids and phages (804 genes). The genes predicted to be of chromosomal origin may constitute a part of the acquired genetic load of various mobile elements. Clustering of KfrA proteins based on their full-length sequences subdivided them into clans of plasmid-related representatives and a few mixed clans of plasmid- and phage-related *kfrA* genes (Fig. 8A). This may suggest a role for KfrA-like proteins also in phage biology. Interestingly, a kind of “mitotic bipolar spindle” has been detected in bacteriophage-infected cells of *Pseudomonas* spp. (55). The filament-forming phage tubulin PhuZ (56) and its roles in the spatial organization of large phage DNA and directed movements of capsids in the cells have been demonstrated (57, 58).

KfrA_RA3_ homologs have been found in *Proteobacteria*, *Firmicutes*, and *Cyanobacteria* (Fig. 8B). Interestingly, the KfrA proteins from IncU (RA3) and IncPβ (R751) were found in the separate but closely related clans, whereas KfrA of RK2, the first identified as an alpha-helical myosin/kinesin-like protein of plasmid origin (26), was not included in this analysis due to the search parameters (see Materials and Methods). Another previously studied KfrA of pPOP15.9 from Paenibacillus popilliae, phylum *Firmicutes* (59) has been clustered together with KfrA_R751_. Notably, KfrA_RK2_ recognizes and binds to an imperfect palindromic motif of 24 nt (26), whereas both KfrA_R751_ and KfrA_POP15.9_ recognize iterated motifs similarly to KfrA_RA3_ (23, 59).

Detailed analysis showed that the closest KfrA_RA3_ homologs are present mainly on IncU plasmids but also on other replicons isolated from *Gammaproteobacteria* (mainly *Aeromonas* spp. and *Enterobacteriaceae*) as well as from unidentified hosts from aquatic samples (60). N-terminal domains of KfrA proteins in this group are almost identical, but proteins differ in the number of 35-amino-acid iterations in the C-terminal tails, from 2 (pMBU17; KC170284) to 15½ repeats (Aeromonas salmonicida pASal5; contig AMQG02000064.1; WP_034284114.1) (Fig. S3A). The second part of the KfrA_RA3_ clan gathered proteins that retained only the N-terminal DNA binding domain but lacked homology in the C-terminal alpha-helical domains (Fig. S3B). These KfrAs were identified in a much wider spectrum of hosts. e.g., Erwinia amylovora pEL60, Staphylococcus equorum, *Halomonas* spp., *Vibrio* spp., and various other marine species. The significance of conservation of KfrAs domains and their roles in plasmid-host interactions require further studies.

## MATERIALS AND METHODS

### Bacterial strains and growth conditions.

The following Escherichia coli strains were used: BL21(DE3) [F^−^ *ompT hsdS*_B_(r_B_^−^ m_B_^−^) *gal dcm* (DE3)] (Novagen), BTH101 [F^−^ *cya-99 araD139 galE15 galK16 rpsL1* (Sm^r^) *hsdR2 mcrA1 mcrB1*] (30), C600 [*thr-1 leu-6 thi-1 lacY1 supE44 ton21 galK*] (61), DH5α [F^−^ ϕ80d*lacZ*ΔM15 *recA1 endA1 gyrA96 thi-1 hsdR17*(r_K_^−^ m_K_^+^) *supE44 relA1 deoR* Δ(*lacZYA-argF*)*U169*], EC1250 [F^−^ *araD139* Δ(*lacZYA-argF*)*U169 rpsL150 relA1 deoC1 ptsF25 rbsR flbB5301 trp-1*] (62), and S17-1 [*recA pro hsdR* RP4-2-Tc::Mu-Km::Tn*7*] (63). The rifampin-resistant mutants Agrobacterium tumefaciens LBA1010 and Paracoccus aminovorans JCM 7685 (64) (kindly supplied by D. Bartosik, University of Warsaw, Poland), Cupriavidus necator (formerly Ralstonia eutropha) JMP228 (kindly supplied by K. Smalla (Julius Kühn-Institut, Federal Research Institute for Cultivated Plants, Germany), and Pseudomonas putida KT2442 (kindly supplied by C. M. Thomas, University of Birmingham, United Kingdom) were used as recipients in conjugation.

Bacteria were generally grown in L broth (65) or on L agar (L broth with 1.5% [wt/vol] agar) at 37°C (E. coli strains apart from BTH101) or at 28°C (A. tumefaciens, C. necator, *P. aminovorans*, P. putida and E. coli BTH101). MacConkey agar base (BD Difco) supplemented with 1% maltose was used in the bacterial adenylate cyclase-based two-hybrid system (BACTH). When required, the media were supplemented with X-Gal (5-bromo-4-chloro-3-indolyl-β-d-galactopyranoside) (40 μg ml^−1^) for blue/white screening, IPTG (isopropyl-β-d-thiogalactopyranoside) for *tacp* induction, or the appropriate antibiotic(s): chloramphenicol (Cm) (10 μg ml^−1^ for E. coli, 50 μg ml^−1^ for A. tumefaciens, 150 μg ml^−1^ for C. necator), kanamycin (Km) (50 μg ml^−1^ for E. coli, 20 μg ml^−1^ for *P. aminovorans* and P. putida), penicillin (Pn) (sodium salt) (150 μg ml^−1^ in liquid media and 300 μg ml^−1^ for agar plates), or rifampin (Rif) (100 μg ml^−1^).

### Plasmid DNA isolation, analysis, amplification, and manipulation.

DNA manipulations were performed according to standard methods (66) and instructions provided by manufacturers of kits used for plasmid isolation, enzymatic reactions (PCR, restriction digestion, ligation, and Gibson assembly), and DNA purification. Complementary oligonucleotides to be cloned in plasmids were first annealed by heating to 95°C and slow cooling. Inserts in all new plasmid constructs were verified by sequencing at the Laboratory of DNA Sequencing and Oligonucleotide Synthesis, Institute of Biochemistry and Biophysics, Polish Academy of Sciences. Plasmids used in this study are listed in Table 1. Oligonucleotides are presented in Table 2.

**TABLE 1:** Plasmids used in this study^*a*^

Designation	Relevant features or description (reference)
Plasmids provided by others	
pAKB2.55	pGBT30 with *kfrC* without a stop codon (IBB collection)
pAKB4.70	pPT01 with *kfrAp* (IBB collection)
pAKB15.40	pLKB2 with *cyaA*T25-*incC* (33)
pAKB15.40N	pKGB5 with *incC-cyaA*T25 (33)
pAKB15.50	pLKB2 with *cyaA*T25-*korB* (33)
pAKB15.50N	pKGB5 with *korB-cyaA*T25 (33)
pAKB16.40	pLKB4 with *cyaA*T18-*incC* (33)
pAKB16.40N	pKGB4 with *incC-cyaA*T18 (33)
pAKB16.50	pLKB4 with *cyaA*T18-*korB* (33)
pAKBB16.50N	pKGB4 with *korB-cyaA*T18 (33)
pESB1.20	pBGS18 with *orf02p-orf02-klcAp-klcA-orf04-korCp-korC-kfrC-kfrAp-kfrA*; part I of the RA3 stability module (WT cassette) (18)
pESB2.29	pUC18 with ′*kfrA-korAp-korA-incC-korB-orf11-mpr-parS*; part II of the RA3 stability module (WT partition cassette) (18)
pESB36	Mini-RK2 derivative; *oriT*_RK2_ *repAp-lacZ lacI*^q^-*tacp-korB*_RK2_ Cm^r^ Km^r^; unstable; RK2 mobilizable vector (18)
pESB36.35	pESB36 with synthetic **RA3 stability module WT**; RA3 coordinates, 2066–9797 (18)
pESB36.44	pESB36 with synthetic RA3 stability module Δ(kfrAp*-*kfrA*); RA3 coordinates, 2066–4833 and 5942–9797 (18)
pET28a(+)	*ori*_MB1_, Km^r^, T7p *lacO*, His_6_ tag, T7 tag; expression vector (Novagen)
pETmod	pET28a(+) derivative deprived of the T7 tag (80)
pGBT30	oriV_MB1_ Ap^r^ *lacI*^q^; *tacp* expression vector (25)
pKAB21	pUC19 derivative with *his_6_-mcs*-FLAG; allows in-frame attachment of *his_6_* to the 5′ end and/or the FLAG gene to the 3′ end of a gene (IBB collection)
pKAB28	pET28mod with deletion of the *his_6_*-T7 tag and EcoRI site adjacent to RBS (81)
pKGB4	pUT18 derivative with modified MCS (72)
pKGB5	pKNT25 derivative with modified MCS (72)
pKNT25	*ori*_p15_, Km^r^, *lacp*-MCS-*cyaA*T25 (30)
pKT25	*ori*_p15_, Km^r^, *lacp-cyaA*T25-MCS (30)
pKT25-zip	pKT25 derivative encoding CyaAT25 in translational fusion with the leucine zipper of GCN4 (30)
pLKB2	pKT25 with modified MCS (82)
pLKB4	pUT18C with modified MCS (82)
pPT01	*ori*_SC101_, Km^r^, promoterless *xylE*; promoter probe vector (83)
RA3	IncU, Cm^r^ Sm^r^ Su^r^ (16)
pUC18	*ori*_MB1_ *bla* (Pn^r^), cloning vector (84)
pUT18	*ori*_ColE1_ Ap^r^ *lacp*-MCS-*cyaA*T18 (30)
pUT18C	*ori*_ColE1_ Ap^r^ *lacp-cyaA*T18–MCS (30)

Plasmids constructed during this work	
pESB1.55	pBGS18 with *kfrA*_1-177_, PCR fragment amplified with the use of primers 30 and 33 on RA3 template and inserted between EcoRI and SalI restriction sites; RA3 coordinates, 4889–5418
pESB2.61	pUC18 with O_K_1; annealed oligonucleotides 1 and 2 inserted between EcoRI and SalI restriction sites
pESB2.62	pUC18 with O_K_2; annealed oligonucleotides 3 and 4 inserted between SmaI and HindIII restriction sites
pESB2.63	pUC18 with O_K_3; annealed oligonucleotides 5 and 6 inserted between EcoRI and SalI restriction sites
pESB2.64	pUC18 with O_K_4; annealed oligonucleotides 7 and 8 inserted between SmaI and HincII restriction sites
pESB2.65	pUC18 with O_K_5; annealed oligonucleotides 9 and 10 inserted in the SmaI restriction site
pESB2.66	pUC18 with O_K_6; annealed oligonucleotides 11 and 12 inserted in the SmaI restriction site
pESB2.67	pUC18 with O_K_7; annealed oligonucleotide 13 inserted in the BamHI restriction site
pESB2.68	pUC18 with WT O_K_; annealed oligonucleotides 3 and 4 inserted between PsiI and HindIII restriction sites of pESB2.61
pESB5.53	pGBT30 with *tacp-kfrA*_54–177_; RA3 coordinates, 5048–5418^*b*^
pESB5.54	pGBT30 with *tacp-kfrA*_178–355_; RA3 coordinates, 5420–5956^*b*^
pESB5.55	pGBT30 with *tacp-kfrA*_1-177_; RA3 coordinates, 4889–5418^*b*^
pESB5.56	pGBT30 with *tacp-kfrA*_Δ54–177_; RA3 coordinates, 4889–5047 and 5419–5956^*b*^
pESB5.57	pGBT30 with *tacp-kfrA*_54–355_; RA3 coordinates, 5048–5956^*b*^
pESB5.58	pGBT30 with *tacp-kfrA*; RA3 coordinates, 4889–5956^*b*^
pESB5.58.1	pGBT30 with *tacp-kfrA*_N29A_; mutations introduced by Gibson assembly of PCR fragments amplified with primer pairs 37/40 and 39/38 on the pESB1.55 template and then recloned into pESB5.54
pESB5.58.2	pGBT30 with *tacp-kfrA*_E30A_; mutations introduced by Gibson assembly of PCR fragments amplified with primer pairs 37/42 and 41/38 on the pESB1.55 template and then recloned into pESB5.54
pESB5.58.3	pGBT30 with *tacp-kfrA*_Q31A_; mutations introduced by Gibson assembly of PCR fragments amplified with primer pairs 37/44 and 43/38 on the pESB1.55 template and then recloned into pESB5.54
pESB5.58.4	pGBT30 with *tacp-kfrA*_V32A_; mutations introduced by Gibson assembly of PCR fragments amplified with primer pairs 37/46 and 45/38 on the pESB1.55 template and then recloned into pESB5.54
pESB5.58.5	pGBT30 with *tacp-kfrA*_R33A_; mutations introduced by Gibson assembly of PCR fragments amplified with primer pairs 37/48 and 47/38 on the pESB1.55 template and then recloned into pESB5.54
pESB5.58.6	pGBT30 with *tacp-kfrA*_E34A_; mutations introduced by Gibson assembly of PCR fragments amplified with primer pairs 37/50 and 49/38 on the pESB1.55 template and then recloned into pESB5.54
pESB5.58.7	pGBT30 with *tacp-kfrA*_R35A_; mutations introduced by Gibson assembly of PCR fragments amplified with primer pairs 37/52 and 51/38 on the pESB1.55 template and then recloned into pESB5.54
pESB5.58.8	pGBT30 with *tacp-kfrA*_M36A_; mutations introduced by Gibson assembly of PCR fragments amplified with primer pairs 37/54 and 53/38 on the pESB1.55 template and then recloned into pESB5.54
pESB5.58.9	pGBT30 with *tacp-kfrA*_G37A_; mutations introduced by Gibson assembly of PCR fragments amplified with primer pairs 37/56 and 55/38 on the pESB1.55 template and then recloned into pESB5.54
pESB5.58.10	pGBT30 with *tacp-kfrA*_G38A_; mutations introduced by Gibson assembly of PCR fragments amplified with primer pairs 37/58 and 57/38 on the pESB1.55 template and then recloned into pESB5.54
pESB5.58.11	pGBT30 with *tacp-kfrA*_G39A_; mutations introduced by Gibson assembly of PCR fragments amplified with primer pairs 37/60 and 59/38 on the pESB1.55 template and then recloned into pESB5.54
pESB5.58.12	pGBT30 with *tacp-kfrA*_S40A_; mutations introduced by Gibson assembly of PCR fragments amplified with primer pairs 37/62 and 61/38 on the pESB1.55 template and then recloned into pESB5.54
pESB5.58.13	pGBT30 with *tacp-kfrA*_L41A_; mutations introduced by Gibson assembly of PCR fragments amplified with primer pairs 37/64 and 63/38 on the pESB1.55 template and then recloned into pESB5.54
pESB5.58.14	pGBT30 with *tacp-kfrA*_S42A_; mutations introduced by Gibson assembly of PCR fragments amplified with primer pairs 37/66 and 65/38 on the pESB1.55 template and then recloned into pESB5.54
pESB5.58.15	pGBT30 with *tacp-kfrA*_H43A_; mutations introduced by Gibson assembly of PCR fragments amplified with primer pairs 37/68 and 67/38 on the pESB1.55 template and then recloned into pESB5.54
pESB5.58.16	pGBT30 with *tacp-kfrA*_I44A_; mutations introduced by Gibson assembly of PCR fragments amplified with primer pairs 37/70 and 69/38 on the pESB1.55 template and then recloned into pESB5.54
pESB5.58.17	pGBT30 with *tacp-kfrA*_S45A_; mutations introduced by Gibson assembly of PCR fragments amplified with primer pairs 37/72 and 71/38 on the pESB1.55 template and then recloned into pESB5.54
pESB5.58.18	pGBT30 with *tacp-kfrA*_P46A_; mutations introduced by Gibson assembly of PCR fragments amplified with primer pairs 37/74 and 73/38 on the pESB1.55 template and then recloned into pESB5.54
pESB5.58.19	pGBT30 with *tacp-kfrA*_V47A_; mutations introduced by Gibson assembly of PCR fragments amplified with primer pairs 37/76 and 75/38 on the pESB1.55 template and then recloned into pESB5.54
pESB5.58.20	pGBT30 with *tacp-kfrA*_M48A_; mutations introduced by Gibson assembly of PCR fragments amplified with primer pairs 37/78 and 77/38 on the pESB1.55 template and then recloned into pESB5.54
pESB5.58.21	pGBT30 with *tacp-kfrA*_R49A_; mutations introduced by Gibson assembly of PCR fragments amplified with primer pairs 37/80 and 79/38 on the pESB1.55 template and then recloned into pESB5.54
pESB5.58.22	pGBT30 with *tacp-kfrA*_E50A_; mutations introduced by Gibson assembly of PCR fragments amplified with primer pairs 37/82 and 81/38 on the pESB1.55 template and then recloned into pESB5.54
pESB5.58.23	pGBT30 with *tacp-kfrA*_W51A_; mutations introduced by Gibson assembly of PCR fragments amplified with primer pairs 37/84 and 83/38 on the pESB1.55 template and then recloned into pESB5.54
pESB5.58.24	pGBT30 with *tacp-kfrA*_R52A_; mutations introduced by Gibson assembly of PCR fragments amplified with primer pairs 37/86 and 85/38 on the pESB1.55 template and then recloned into pESB5.54
pESB5.60	pGBT30 with *tacp-kfrA*_1–53_^*b*^
pESB6.53	pETmod with T7p-*kfrA*_54–177_; EcoRI-SalI fragment from pESB5.53
pESB6.54	pETmod with T7p-*kfrA*_178–355_; EcoRI-SalI fragment from pESB5.54
pESB6.55	pETmod with T7p-*kfrA*_1-177_; EcoRI-SalI fragment from pESB5.55
pESB6.56	pETmod with T7p-*kfrA*_Δ54–177_; EcoRI-SalI fragment from pESB5.56
pESB6.57	pETmod with T7p-*kfrA*_54–355_; EcoRI-SalI fragment from pESB5.57
pESB6.58	pETmod with T7p-*kfrA*; EcoRI-SalI fragment from pESB5.58
pESB6.58.5	pETmod with T7p-*kfrA*_R33A_; EcoRI-SalI fragment from pESB5.58.5
pESB6.58.10	pETmod with T7p -*kfrA*_G38A_; EcoRI-SalI fragment from pESB5.58.10
pESB6.58.11	pETmod with T7p-*kfrA*_G39A_; EcoRI-SalI fragment from pESB5.58.11
pESB6.58.13	pETmod with T7p-*kfrA*_L41A_; EcoRI-SalI fragment from pESB5.58.13
pESB6.58.16	pETmod with T7p-*kfrA*_I44A_; EcoRI-SalI fragment from pESB5.58.13
pESB6.58.18	pETmod with T7p-*kfrA*_P46A_; EcoRI-SalI fragment from pESB5.58.18
pESB6.60	pETmod with T7p-*kfrA*_1–53_; EcoRI-SalI fragment from pESB5.60
pESB6.89	pETmod with T7p-*kfrA*_178-336_
pESB13.80	pPT01 with WT *kfrAp-xylE*; annealed oligonucleotides 14 and 15 inserted between SphI and BamHI restriction sites
pESB13.81	pPT01 with *kfrAp*_DR1234_-*xylE*; annealed oligonucleotides 16 and 17 inserted between SphI and BamHI restriction sites
pESB13.82	pPT01 with *kfrAp*_DR1234r_-*xylE*; annealed oligonucleotides 18 and 19 inserted between SphI and BamHI restriction sites
pESB13.83	pPT01 with *kfrAp*_DR234_-*xylE*; annealed oligonucleotides 20 and 21 inserted between SphI and BamHI restriction sites
pESB13.84	pPT01 with *kfrAp*_DR25_-*xylE*; annealed oligonucleotides 22 and 23 inserted between SphI and BamHI restriction sites
pESB13.85	pPT01 with *kfrAp*_DR34_-*xylE*; annealed oligonucleotides 24 and 25 inserted between SphI and BamHI restriction sites
pESB13.86	pPT01 with *kfrAp*_DR23_-*xylE*; annealed oligonucleotides 26 and 27 inserted between SphI and BamHI restriction sites
pESB13.87	pPT01 with *kfrAp*_DR3_-*xylE*; annealed oligonucleotides 28 and 29 inserted between SphI and BamHI restriction sites
pESB15	pET28a(+) derivative; allows in frame attachment of *his_6_* to the 3′ of a gene cloned as an EcoRI-HindIII fragment^*b*^
pESB15.90	pESB15 with T7p-*kfrC*^*b*^
pESB36.34	pESB36 with synthetic **RA3 stability module Δ(*kfrA*)-t*_tnp513_*** (*orf02p-orf02-klcAp-klcA-orf04-korCp-korC-kfrC-kfrAp*-t*_tnp513_-korAp-korA-incC-korB-orf11-mpr-parS*); RA3 coordinates, 2066–4882 and 5942–9797
pESB36.42	pESB36 with synthetic **RA3 stability module Δ*kfrC*** (*orf02p-orf02-klcAp-klcA-orf04-korCp-korC-kfrC*′-*kfrAp-kfrA-korAp-korA-incC-korB-orf11-mpr-parS*); RA3 coordinates, 2066–4257 and 4747–9797*d*
pESB36.45	pESB36 with synthetic **RA3 stability module Δ(*kfrC-kfrA*)** (*orf02p-orf02-klcAp-klcA-orf04-korCp-korC-kfrC*′-*korAp-korA-incC-korB-orf11-mpr-parS*); RA3 coordinates, 2066–4257 and 5942–9797d
pESB36.48	pESB36 with synthetic **RA3 stability module *kfrA*-t*_tnp513_*** (*orf02p-orf02-klcAp-klcA-orf04-korCp-korC-kfrC-kfrAp-kfrA*-t*_tnp513_-korAp-korA-incC-korB-orf11-mpr-parS*); RA3 coordinates, 2066–9797
pJKB9	pESB36 with synthetic **RA3 stability module *kfrA*_L41A_-t*_tnp513_*** (*orf02p-orf02-klcAp-klcA-orf04-korCp-korC-kfrC-kfrAp-kfrA*_L41A_-t*_tnp513_-korAp-korA-incC-korB-orf11-mpr-parS*)
pOMB4.13	pLKB4 with *cyaA*T18-*kfrA*^*b*^
pOMB4.15	pLKB4 with *cyaA*T18-*kfrC*^*b*^
pOMB5.1	pLKB2 with *cyaA*T25-*kfrA*_1–53_^*b*^
pOMB5.2	pLKB2 with *cyaA*T25-*kfrA*_54–177_^*b*^
pOMB5.3	pLKB2 with *cyaA*T25-*kfrA*_178–355_^*b*^
pOMB5.4	pLKB2 with *cyaA*T25-*kfrA*_1-177_^*b*^
pOMB5.5	pLKB2 with *cyaA*T25-*kfrA*_Δ54–177_^*b*^
pOMB5.6	pLKB2 with *cyaA*T25-*kfrA*_54–355_^*b*^
pOMB5.13	pLKB2 with *cyaA*T25-*kfrA*^*b*^
pOMB5.15	pLKB2 with *cyaA*T25-*kfrC*^*b*^
pOMB6.14	pKGB4 with *kfrA-cyaA*T18^*b*^
pOMB6.16.1	pKGB4 with *kfrC-cyaA*T18^*b*^
pOMB7.7	pKGB5 with *kfrA*_1–53_-*cyaA*T25^*b*^
pOMB7.8	pKGB5 with *kfrA*_54–177_-*cyaA*T25^*b*^
pOMB7.9	pKGB5 with *kfrA*_178–355_-*cyaA*T25^*b*^
pOMB7.10	pKGB5 with *kfrA*_1-177_-*cyaA*T25^*b*^
pOMB7.11	pKGB5 with *kfrA*_Δ54–177_-*cyaA*T25^*b*^
pOMB7.12	pKGB5 with *kfrA*_54–355_-*cyaA*T25^*b*^
pOMB7.14	pKGB5 with *kfrA-cyaA*T25^*b*^
pOMB7.16.1	pKGB5 with *kfrC-cyaA*T25^*b*^
pOMB8.77	pKAB28 with *his_6_-kfrA kfrC*-FLAG^*b*^

a MCS, multiple-cloning site; RBS, ribosome binding site. A prime symbol indicates a truncated ORF; the position of the symbol indicates a 5′- or 3′-end deletion. Boldface type indicates variants of the RA3 stability module cloned in the test vector. Primer sequences are presented in Table 2.

b A detailed description of plasmid construction is included in the supplemental material.

**TABLE 2:** Oligonucleotides used in this study^*a*^

No.	Designation	Sequence
1	OG1	AATTCGTTGTATTGTATGTATTGTATGTATTGTATTATAAG
2	OD1	TCGACTTATAATACAATACATACAATACATACAATACAACG
3	OG2	AAATACAATACATACAATACAGGA
4	OD2	AGCTTCCTGTATTGTATGTATTGTATTT
5	OG3	AATTCTATGTATTGTATTAAAATACAATACATAG
6	OD3	TCGACTATGTATTGTATTTTAATACAATACATAG
7	OG4	TATGTATTGTATGTATTGTATTAAAATACAATACATA
8	OD4	TATGTATTGTATTTTAATACAATACATACAATACATA
9	OG5	TATGTATTGTATGTATTGTATTAAAATACAATACATACAATACAGG
10	OD5	CCTGTATTGTATGTATTGTATTTTAATACAATACATACAATACATA
11	OG6	GTTGTATTGTATGTATTGTATGTATTGTATTAAAATACAATACATA
12	OD6	TATGTATTGTATTTTAATACAATACATACAATACATACAATACAAC
13	ODGW6	GATCTGTTGTATTGTATGTATTGTATGTATTGTATGTATTGTAACA
14	PrWTG	GTGTATTGTATGTATTGTATGTATTGTATTAAAATACAATACATACAATACAG
15	PrWTD	GATCCTGTATTGTATGTATTGTATTTTAATACAATACATACAATACATACAATACACCATG
16	Pr4pG	GTGTATTGTATGTATTGTATGTATTGTATTAAAATACAATACAataactgacG
17	Pr4pD	GATCCgtcgttatTGTATTGTATTTTAATACAATACATACAATACATACAATACACCATG
18	Pr4tG	GTGTATTGTATGTATTGTATGTATTGTATTAAAATgtAtTgtAataAcTgacG
19	Pr4tD	gatccgtcAgTtatTacAaTacATTTTAATACAATACATACAATACATACAATACACCATG
20	Pr234G	GTTGTATGTATTGTATGTATTGTATTAAAATACAATACAG
21	Pr123D	GATCCTGTATTGTATTTTAATACAATACATACAATACATACAACCATG
22	Pr25NG	GTTGTATGTATTGTAataAaTtatTTAAAATAatAataATACAATACAG
23	Pr25ND	GATCCTGTATTGTATtatTatTATTTTAAataAtTtatTACAATACATACAACCATG
24	Pr2ppF	GTTGTATcaATcaaATGTATTGTATTAAAATACAATACAG
25	Pr2ppR	GATCCTGTATTGTATTTTAATACAATACATttgATtgATACAACCATG
26	Pr2i3G	GTTGTATGTATTGTATGTATTGTATTAAAATcCtAatgAG
27	Pr2i3D	GATCCTcatTaGgATTTTAATACAATACATACAATACATACAACCATG
28	PrDR3G	GTTGTATcaATcaaATGTATTGTATTAAAATAtAActgAcG
29	PrDR3D	GATCCgTcagTTaTATTTTAATACAATACATttgATtgATACAACCATG
30	Ia	cgccaattgagatctgaattc**ATG**ACCATGATTAAGCCTG
31	IbN	GCGGTCGACG*gc*GCCCGCCACTCACGCATA
32	IIa	cggaattc**atg**agCTCTCAAGAGCAATCGGACA
33	IIb	gcggtcga**cta**cGTAAACGACTCGATAGACTGG
34	IIIa	cggaattcat*gagCTCTGATAATGCCACGCTCA
35	IIIb	gcggtcgacggatcc**TTA**CTCTTTGGTGTCGGCTG
36	IbSTOP	gcgtcgac**tta**GGCCCGCCACTCACGCAT
37	WF	TGCGGTATTTCACACCGCATATGGTG
38	WR	AAGCTTGCATGCCTGCAGGTCGACTAC
39	1F2	AGCAAAGCCAACAgcTGAGCAGGTGCGCGAG
40	1R1	CTCAgcTGTTGGCTTTGCTACCCCTTC
41	2F2	AAATGcGCAGGTtCGCGAGCGCATGGGCG
42	2R1	CTCGCGaACCTGCgCATTTGTTGGCTTTGCTAC
43	3F2	AAATGAGgcGGTtCGCGAGCGCATGGGCG
44	3R1	CTCGCGaACCgcCTCATTTGTTGGCTTTGC
45	4F2	AAATGAGCAGGctCGCGAGCGCATGGGCG
46	4R1	CTCGCGagCCTGCTCATTTGTTGGCTTTGC
47	5F2	GCAGGTGgctGAGCGCATGGGCGGCGGCT
48	5R1	CATGCGCTCagcCACCTGCTCATTTGTTG
49	6F2	AGGTGCGCGccCGCATGGGCGGCGGCT
50	6R1	CCCATGCGggCGCGCACCTGCTCAT
51	7F2	GCGCGAGgcCATGGGCGGCGGCTCTTTGT
52	7R1	GCCGCCCATGgcCTCGCGCACCTGCTCAT
53	8F2	CGAGCGCgccGGCGGCGGCTCTTTGTC
54	8R1	GCCGCCGCCggcGCGCTCGCGCACCTG
55	9F2	GAGCGCATGGcCGGCGGCTCTTTGTC
56	9R1	AGCCGCCGgCCATGCGCTCGCGCAC
57	10F2	GCGCATGGGCGcCGGCTCTTTGTCTCA
58	10R1	AGAGCCGgCGCCCATGCGCTCGCGCA
59	11F2	TGGGCGGCGcCTCTTTGTCTCATATC
60	11R1	GACAAAGAGgCGCCGCCCATGCGCTCG
61	12F2	GCGGCGGCgCcTTGTCTCATATCTCG
62	12R1	TGAGACAAgGcGCCGCCGCCCATGCGC
63	13F2	CTgcGTCTCATATCTCaCCGGTTATGCGTGAGT
64	13R1	GGtGAGATATGAGACgcAGAGCCGCCGCCCATG
65	14F2	TGgCTCATATCTCaCCGGTTATGCGTGAGT
66	14R1	GGtGAGATATGAGcCAAAGAGCCGCCGCCCATG
67	15F2	TCTTTGTCTgcTATCTCaCCGGTTATGCGTGAGT
68	15R1	GtGAGATAgcAGACAAAGAGCCGCCGCCCATG
69	16F2	GCTCTTTGTCgCATgcCTCGCCGGTTATGCGT
70	16R1	GAGgcATGcGACAAAGAGCCGCCGCCCATG
71	17F2	TTTGTCTCATATCgCaCCGGTTATGCGTGAGT
72	17R1	CATAACCGGtGcGATATGAGACAAAGAGC
73	18F2	CTCATATCTCcgCGGTTATGCGTGAGTG
74	18R1	ATAACCGcgGAGATATGAGACAAAGAG
75	19F2	CGCCGGcTATGCGTGAGTGGCGGGCCTC
76	19R1	CACTCACGCATAgCCGGCGAGATATGAGAC
77	20F2	CTCGCCGGTTgcaCGTGAGTGGCGGGCCT
78	20R1	ACTCACGtgcAACCGGCGAGATATGAGAC
79	21F2	TATGgcTGAGTGGCGcGCCTCTCAAGAGCAATC
80	21R1	GGCgCGCCACTCAgcCATAACCGGCGAGATA
81	22F2	TGCGTGcGTGGCGcGCCTCTCAAGAGCAATC
82	22R1	GAGGCgCGCCACgCACGCATAACCGGCGAGAT
83	23F2	GCGTGAGgccCGGGCCTCTCAAGAGCAATC
84	23R1	AGAGGCCCGggcCTCACGCATAACCGGCGA
85	24F2	AGTGGGCGGCgTCTCAAGAGCAATCGGAC
86	24R1	TCTTGAGAcGCCGCCCACTCACGCATAAC
87	OKFRCD2	tcgacggtaccAGCGGCTTCACCGCT
88	OKFRCG2	CTAGAGCGG**TGA**AGCCGCTggtaccg
89	IR	cggtcgacGGCCCGCCACTCACGCATAA
90	IIR	cggtcgacGGTAAACGACTCGATAGACT
91	IIIbstop	gcggtcgaccccgggCTCTTTGGTGTCGGCTGC
92	Ttnp513Eco	aattcagatctTGAAGCCCCAACTGTTATCAGTTGGGGCTTTTTCTTGTCTGTTTt
93	Ttnp513Bcl	gatcaAAACAGACAAGAAAAAGCCCCAACTGATAACAGTTGGGGCTTCAagatctg
94	STOPSal	Gtaactagttaggtcgacctaactagttac
95	*kfr*CrbsF	cggaattcagatcta*aggagG*AAACC**ATG**ACCGAACATAA
96	*kfr*Cstop	cggtcgacCCGCTCTAGATCGTCTTCAT
97	KFRCBSD	tcgacaagcttCCGCT
98	KFRCBSG	CTAGAGCGGaagcttg
99	OPETD	GATCgtgcAGC
100	OPETG	CATGGCtgcac

a Start and stop codons are in bold, the introduced restriction sites or overhangs are underlined, nucleotides not complementary to the template are in lowercase letters, and an additional Shine-Dalgarno sequence is in italics.

### Plasmid construction.

**(i) Construction of *kfrA* deletion mutants.** The *kfrA* fragments *kfrA*_1–53_, *kfrA*_54–177_, *kfrA*_178–355_, and *kfrA*_1-177_ were generated by PCR on the RA3 template with pairs of primers designed in such a way as to provide start (ATG) and stop (TAA) codons in encoded proteins and appropriate restriction sites to enable joining of the fragments in translational fusions without a change in the encoded amino acid sequence (see the description in the supplemental material). The *kfrA*_178-336_ allele is a *kfrA*_178–355_ derivative in which the HincII-SalI fragment was replaced with self-annealed oligonucleotide 94, providing a stop codon downstream of a K336 codon. *kfrA* and its truncated alleles were first cloned into pUC18 or pBGS18, verified by sequencing, and subsequently recloned as EcoRI-SalI fragments into the expression vector pGBT30 or pET28mod.

**(ii) Construction of KfrA alanine substitution mutants.** The Gibson assembly technique (67) was used to construct plasmids with *kfrA* alleles encoding proteins with particular putative HTH residues replaced by alanines. First, two PCR fragments amplified on the pESB1.55(*kfrA*_1-177_) template and pBGS18 linearized with NdeI and HindIII endonucleases were assembled using Gibson assembly master mix (New England BioLabs). PCR primers provided 19-bp overlaps at the junction regions, nucleotide substitutions encoding an alanine codon in the desired locus, and a new restriction site (without changing the amino acid sequence in the final product of gene expression) to facilitate postcloning plasmid screening. Next, the *kfrA*_1-177_ alleles were cloned as EcoRI-Eco105I fragments between EcoRI and Ecl136II restriction sites of pESB5.54(*kfrA*_178–355_) (final plasmids are listed in Table 1). Subsequently, selected *kfrA* mutant alleles were recloned as EcoRI-SalI fragments into pET28mod.

**(iii) Construction of RA3 stability module variants.** The synthetic RA3 stability module was reconstructed previously (18) from two parts (part 1 including *orf02-klcA-orf04-korC-kfrC-kfrA* cloned into pESB1.20 and part 2 including *korA-incC-korB-orf11-parS* cloned into pESB2.29) obtained by a multistep procedure of joining restriction and PCR-amplified fragments, encoding particular RA3 regions, into the high-copy-number vectors. This strategy was then applied to construct the RA3 stability module variants deprived of the *kfr* genes and/or *kfrAp* as well as to introduce the heterologous transcriptional terminator t*_tnp513_* on annealed oligonucleotides 92 and 93 upstream of the *korA* promoter. After thorough verification of DNA sequence, especially at junction points, variants of the stability module were recloned into the unstable BHR vector pESB36 (18).

### Bacterial transformation and conjugation.

Competent cells of E. coli were prepared by the standard CaCl_2_ method (66).

E. coli S17-1 harboring pESB36 or its derivative with an RA3 stability module variant was used as a donor in conjugation with the chosen Rif^r^ strain (A. tumefaciens, C. necator, *P. aminovorans*, or P. putida) as described previously (18). Briefly, aliquots of 100 μl of stationary-phase cultures of the donor and recipient strains, washed previously with L broth, were mixed on an L agar plate and incubated overnight at 28°C. Bacteria were washed off the plate, and serial dilutions were plated on the appropriate selective solid medium.

### BACTH system.

Dimerization of KfrA and KfrC, as well as interactions of Kfr proteins with IncC or KorB *in vivo*, was analyzed using the bacterial two-hybrid system (30) in the E. coli BTH101 *cyaA* strain. Genes encoding the Kfr proteins or their truncated derivatives were cloned into the BACTH vectors to create translational fusions with CyaAT18 (pKGB4 and pLKB4) or CyaAT25 (pKGB5 and pLKB2) fragments via the N or C terminus, respectively. Construction of derivatives of BACTH vectors is described in detail in the supplemental material. Pairs of the appropriate plasmids were cotransformed into E. coli BTH101 *cyaA*, and transformants were selected on L agar supplemented with kanamycin, penicillin, and 0.15 mM IPTG. Bacteria were incubated for approximately 48 h, and randomly chosen transformants were restreaked on selective MacConkey medium with 1% maltose and used in the β-galactosidase assay (68). Reconstitution of the CyaA activity due to the interactions between analyzed proteins led to the activation of *mal* and *lac* operons in E. coli, manifested by the formation of purple colonies on maltose containing solid medium and an increase of β-galactosidase activity in extracts of liquid cultures. One unit of β-galactosidase is defined as the amount of enzyme needed to convert 1 μmol of *o*-nitrophenyl-β-d-galactopyranoside (ONPG) to *o*-nitrophenol and d-galactose in 1 min under standard conditions.

### Protein purification by immobilized metal ion affinity chromatography.

E. coli BL21(DE3) cells carrying pET28mod derivatives encoding N-terminally His_6_-tagged KfrA, its truncated or mutant variant, or KfrC were grown until an optical density at 600 nm (OD_600_) of 0.6 was reached. Protein overproduction was induced with 0.5 mM IPTG for 3 h. A 200-ml portion of the culture was centrifuged, and harvested cells were broken by ultrasonic treatment in 10 ml of sonication buffer (50 mM sodium phosphate buffer [NaP_i_] pH 8.0, 300 nM NaCl), supplemented with 1 mg ml^−1^ lysozyme and protease inhibitor cocktail (Sigma-Aldrich). The lysate was cleared by centrifugation and loaded onto the Protino Ni-TED 1000 column with a dry silica-based resin with TED (tris-carboxymethyl ethylene diamine) as a chelating group (Macherey-Nagel) preequilibrated with sonication buffer. Bound His_6_-tagged proteins were washed with 10 ml of washing buffer (50 mM NaP_i_ [pH 8.0], 300 mM NaCl, 10% glycerol) and then eluted with elution buffer (50 mM NaP_i_ [pH 8.0], 300 mM NaCl, 10% glycerol, 300 mM imidazole [for KfrA] or imidazole gradient up to 300 mM [for KfrC]) in 0.5-ml fractions. The eluate was dialyzed overnight against dialysis buffer (50 mM NaP_i_ [pH 7.5], 100 mM NaCl). The protein purification was monitored by SDS-PAGE using a PhastSystem (Pharmacia). Protein concentration was determined using the Bradford method (69) or SynergyH4 Hybrid reader at 280 nm.

### Cross-linking with glutaraldehyde.

Purified His_6_-tagged KfrA or its truncated derivatives (0.1 mg ml^−1^) were used in reactions with increasing concentrations (0 to 0.05%) of glutaraldehyde as described previously (70). Proteins were analyzed by SDS-PAGE on polyacrylamide gels of the appropriate concentration (12%, 15%, or 18%) and visualized by Coomassie staining (66).

### Analysis of protein-DNA interactions *in vitro* by EMSA.

DNA of a pUC18 derivative with the KfrA binding site (O_K_) or its variants in DR number and/or spatial arrangement was digested with EcoRI and NdeI restriction nucleases to produce two fragments: a shorter one (approximately 300 bp) carrying analyzed sequence and a longer one (2,420 bp) with the remaining part of the plasmid, used as a competitor. Digested DNA (10 nM) was mixed with increasing concentrations of His_6_-KfrA or its truncated mutant variants in binding buffer (50 mM Tris-HCl [pH 8.0], 10 mM MgCl_2_, 50 mM NaCl) in a final volume of 20 μl and incubated at 37°C for 30 min. The samples were analyzed on 1.5% [wt/vol] agarose gel in 0.5× Tris-borate-EDTA (TBE) buffer (66). The gel was stained with ethidium bromide, and DNA bands were visualized using UV light. Images were analyzed with ImageJ 1.52n (71).

### Coimmunoprecipitation.

Coimmunoprecipitation of KfrA with KfrC was done by a modified method described previously (72). E. coli BL21(DE3) was transformed with pOMB8.77(T7p-*his_6_-kfrA kfrC*-FLAG) or pESB6.58(T7p-*his_6_-kfrA*), which served as a control. Overnight cultures of the transformants were diluted 1:50 into fresh medium, and after 1 h of growth, protein overproduction was induced with 0.5 mM IPTG for another 4 h. The cultures were incubated with formaldehyde (1% [vol/vol]) at room temperature with gentle agitation for 30 min. After sonication of pelleted cells, the immunoprecipitation procedure proceeded with anti-FLAG antibodies (Invitrogen) as described in the supplemental material. Western blot analysis of the initial cell extracts and the coimmunoprecipitated samples with the use of anti-His antibodies (Invitrogen), diluted 1:2,000, was carried out after proteins separation by SDS-PAGE and transfer onto a nitrocellulose membrane.

### Removal of the His_6_ tag from KfrA.

His_6_ tag was cleaved off KfrA with thrombin using the thrombin cleavage capture kit (Novagen) according to the manual. The protease was then removed on streptavidin agarose (included in the kit), and the cleavage efficiency was monitored by Western blotting with the primary monoclonal antibody mouse HIS.H8 (Thermo Scientific) and secondary polyclonal goat anti-mouse antibodies conjugated with horseradish peroxidase (HRP; Dako Denmark A/S) and visualized using a Clarity Western enhanced chemiluminescence (ECL) substrate (Bio-Rad) with a Synergy G-Box chemiluminescent detection system (Syngene).

### MST measurements.

His_6_-KfrC was labeled using the RED-Tris-NTA 2nd-generation dye (NanoTemper Technologies) according to the manual. Labeled His_6_-KfrC (50 nM) was incubated with decreasing concentrations of KfrA (from 500 nM to 0.0153 nM) in the MST binding buffer (50 mM KP_i_ [pH 7.4], 300 mM NaCl, 0.05% Tween 20) for 5 min at 25°C. Reaction mixtures were centrifuged at 15,000 × g for 5 min, and supernatants were loaded into Monolith NT.115 series premium capillaries. The MST fluorescence signal was measured over time at 37°C under medium (40%) MST power and 100% LED (light-emitting diode) power with the Monolith NT.115 instrument (NanoTemper Technologies, Germany). The change in the thermophoresis corresponds to the change in the normalized fluorescence (ΔF_norm_), which is defined as F_hot_/F_cold_ (the ratio between the average fluorescence in the hot region [start, 4 s; end, 5 s] and the cold region [start, −1 s; end, 0 s]). The dose-response fit *K_d_* (dissociation constant) and *K_d_* confidence (in nanomolar units) were calculated from four independent experiments: *K_d_* = [L][P]/[LP], where [L], [P], and [LP] represent molar concentrations of the proteins and the protein complex.

### Determination of catechol 2,3-dioxygenase activity (XylE).

E. coli C600 carrying an appropriate pPT01 derivative was transformed with either the pGBT30 (*tacp*) expression vector or pESB5.58 (*tacp-kfrA*_RA3_). XylE activity (73) was assayed spectrophotometrically in cleared extracts of cells collected from logarithmically growing cultures without and with 0.5 mM IPTG, as described previously (18). One unit of the catechol 2,3-oxygenase activity is defined as the amount of enzyme needed to convert 1 μmol of catechol to 2-hydroxymuconic semialdehyde per mg of protein in 1 min. Protein concentration was determined using the Bradford method (69). Experiments were performed in triplicate, and the mean values with standard deviations are reported.

### Plasmid stability assays.

The stability of pESB36 or its derivative in E. coli EC1250, A. tumefaciens LBA1010 Rif^r^, *P. aminovorans* JCM 7685 Rif^r^, P. putida KT2442 Rif^r^, or C. necator JMP228 Rif^r^ was tested as described previously (18). Briefly, stationary-phase cultures grown under antibiotic selection were diluted 10^5^-fold in fresh medium (without antibiotic) and cultivated for approximately 20 generations. In parallel, diluted cultures were plated on L agar supplemented with X-Gal. Culture refreshing and plating procedures were repeated every 20 generations for up to 60 generations. Plasmid retention was calculated as a percentage of blue colonies. The plasmid loss rate (LR) per generation (percent) was calculated using the following formula:

$$\left(1-\sqrt[{n}]{\frac{F_{f}}{F_{i}}}\right)\times 100$$

where *n* is number of generations elapsed, *F_i_* is the fraction of cells containing the plasmid at the initial time point, and *F_f_* is the fraction of cells containing the plasmid at the final time point. For each strain, stability experiments were performed in triplicate starting from three separate colonies.

### Modeling and sequence analysis.

The HTH domain of KfrA was detected using Meta-BASIC (74) homology detection method. Residue conservation was assessed based on PSI-BLAST (75) hits (nonredundant [NR] database, three iterations, inclusion threshold E value of 0.001) aligned using Mafft (76). Secondary structures were predicted using Psipred (77). Comparative modeling was performed using Modeller (78) based on the structure of KorA (PDB code 2N6G) (29), which served as a template identified by Meta-BASIC. KfrA-DNA docking was performed using HADDOCK 2.4 (79) and manually refined. The model of the KfrA dimer was derived based on the structure of TubR plasmid segregation protein (PDB code 3M8E) (54). The model was relaxed using the Rosetta relax protocol. All structures were rendered using PyMOL (PyMOL Molecular Graphics System, version 2.0; Schrödinger, LLC). The clustering of protein sequences was performed using CLANS (85).

## Supplementary Material

Supplemental file 1
