# Supplementary material for: Unique Properties of the Alpha-Helical DNA-Binding Protein KfrA Encoded by the IncU Incompatibility Group Plasmid RA3 and Its Host-Dependent Role in Plasmid Maintenance
Source: Appl Environ Microbiol. 2021 Jan 4;87(2):e01771-20. doi: 10.1128/AEM.01771-20 (PMC7783346; doi:10.1128/AEM.01771-20)
Supplement: Supplemental file 1 [file AEM.01771-20-s0001.pdf]

Figure S1

```
                                             HHHHHHHHHHHHH       HHHHHHHH     HHHHHHHHHHHHHHH
ABD64835.1 RA3        Ah    (  7) MKEKIVRAAESLRSEGVAKPTNEQVRERMGG-GSLSHISPVMREWRASQEQS (297)
WP_010890107.1 R751   Ea    (  3) SKEQIFAVADELDAA-GQNPTLANVRKQLGS-GSFTTISEAMNEWRARKASQ (289)
WP_106486033.1        Ec    ( 35) TVRRIEDAAATLIAAGTPNPTNEQVRQHLGG-GSLSHISPVMRAFRARQREQ (119)
MBN56213.1            Ob    (  6) IKERIVEAANALVAEGNENPTNEQVRERMGS-GSLSHISPVMREWRESQKAR (144)
ECA5366687.1          Se    (  7) MKEKIVRAAESLRAEGVAKPTNEQVRERMGG-GSLSHISPVMREWRASQEQS (129)
PHS21351.1            Hs    (  6) IRDRILDAAEELVAEGIDRPTNDQVRERLGG-GSLSHISPVMREWRENMKVS (292)
WP_159185344.1        Kp    (  6) IRARIEAAAVALVSEGVDNPTNEQVRQKLGS-GSLSHISPVMREWRQKRREQ (154)
KIU77917.1            Vp    (  4) -KQEIIDAAEKLAAEGV-NPSMQAVRERLGG-GSFATISPVLREWKENREAT ( 19)
WP_147252233.1        Mp    ( 85) -EDRIIEAAEKLEAEGV-NPTQVNVRDALGG-GSFATIGPVLKKWKESKKED (250)
WP_075882038.1        HsM   (  4) -KEDIFNVAERLAAEGT-NPTQTAVREALGG-GSFATIGPALKEWKDAQRED (161)
WP_051226028.1        Mc    ( 40) -VEQVHRAADELAGQGI-NPTQVAVRKQLGG-GSFSTIGEALKSWRAMRDTN (199)
TGP43006.1            bM    (  4) -REQILETASKLAEQGI-KPTQTNVRESLGG-GSFTTISEVLREWRQDQDQT ( 92)
WP_160002967.1        Mb1   (  4) -IQQIHATADQLQEQGI-KPTLAEVRKILGG-GSFTTISEAMKSWRQDNQEE (138)
EDX0374294.1          Se    ( 16) ILCRIEDAAAALIADGTPNPANVRGRDNPGG-GSLATILPVMRAYRVRLRER (  3)
EAN5736141.1          Se    (  4) -TEQIMKAADELNQEGQ-NPTLARVRKKLGG-GSFTTISEVMIEWRALKARS ( 13)
MQT82934.1            Ph    (  4) -KEMIWEAADELDADGT-KPTLANVRKRLGGVGSFTTIQEAMSEWKNRKQQE ( 37)
KMY85731.1            CPc   (  4) -RDAIWQVADALAAESV-KPTLAAVRKKIGG-GSFTTIQEAMAEWKARRQQI (134)
KFA35857.1            Xv    (  4) -REQIWAAADNLDAAGQ-NPTLVAVRKVVGG-GSFSTIQDAMAEWKARRAAK (  8)
WP_122181186.1        C     (  4) -KDQIWTAADELDGAGK-PPTLAAVRKAVGG-GSFTTIQDAMTEWKAKRQTK (147)
EWS66565.1            HsT   (  4) -KEQIFQAADELDAMGK-NPTLAEVRKLVGG-GSFTTISEAMNEWRARKSAQ (292)
PLO94891.1            Kpn   (  4) -KEQIFEAADQLAAAGQ-KPTLEAIRQ-ITG-GSYTTISPVLNEWKARQAAQ ( 29)
EDH5293470.1          Se    (  4) -KDQVFRVADELDAAGQ-KPTLAAVRKALGG-GSFTTISEAMNEWRAHRAAQ (169)
WP_002706521.1        Tn    (  4) -KEQIFHAADQLAESGE-SPTLANVRKALGG-GSFTTISEAMNEWKAKQQAT (273)
WP_138860543.1        TsU   (  4) -KQDILAVADALDAEGV-KPTLAAVRKKLGG-GSFSTISEAMKEWKADHASA (254)
OQW85873.1            Rf    (  4) -KDDIFLAANELDALGQ-SPTLAAVRKHLGG-GSFTTISEAMKEWKSKKTVK (155)
WP_017171842.1        Xp    (  4) -RDQILKAADELDAEGI-KPTLNNVRKRLGG-GSFTTISDVMTEWKQRQLQK (159)
WP_131469984.1        Xc    (  4) -KDQIWKIADQLDADGV-KPTLSAVRKKLGS-GSYTTIQDAMNEWKQRKLQK (160)
WP_090142701.1        LsD   (  4) -TARIFEIADELDAAGH-NPTLASVRKALGG-GSYTTISQAMTEWRARKAAK (149)
WP_140110024.1        Vpa   (  4) -QEKIWKIADELDAAGK-KPTLAAIRKELNG-GSYTTISEAMKEWRAKQAND (300)
WP_013028091.1        Nh    (  4) -KEDIWKAADELEIAGQ-APTLAAVRKALGG-GSFTTISEAMKEWRAKRVRE (193)
GAC44101.1            PpA1  (  8) -KEQIWAAAEKLLQEGK-SPTLAAVRGVVGG-GSYTTISEAMSEFRAVQEKT ( 39)
WP_075657552.1        PsB   (  4) -KQDIWRCADELDAEGI-RPTLAAVRKKLGS-GSYTTISEAMAEWKNRKQAS (157)
WP_128102775.1        PsD   (  4) -KEQIFTVADTLDSEGI-NPTLANVRKRLGS-GSFSTISEYMAEWREERKDT (217)
WP_082844505.1        Rm    ( 23) -RDDIWAVADRLDAEGK-APTLAAVRKALGS-GSFTTISEAMREWHQRRKEK (144)
WP_093036880.1        Tr    ( 17) -HEQIIEVADALVAAGQ-NPTMASVRKALGG-GSFTTISEALKAWRQTRQEE (253)
TAK94307.1            Pgb   (  1) -KTKIFAICEQLQKTGV-KPTLEKVREALGG-GSFSTINPILKEWKEQQSTS (255)
WP_067517600.1        Ea    (  4) -RDTVFTAAEAILVRGE-KPTNEKVRNELGS-GSFSDIGPHFREWRDRQSSH (135)
WP_083571023.1        HsQ   ( 32) -NERIHEIANQLSDKGI-KPTLNAVRNELGS-GSFSTIGQAMKIWREAQETD (139)
OTE97472.1            CsD   (  1) -KERVFTACNQLTKQGV-KPTLAQVRNALGG-GSFSTISPFFRQWKEDRMTH ( 83)
OYV18940.1            MbN   (  1) -KKRIFNICNQLVKQNI-KPTLLRVRSELGG-GSFSTINPIFKQWKEDSRTR ( 96)
WP_162926463.1        CbB   (  8) KIKKIHEAADAIVADGNEAPTNDEVREKAGG-GSISDISFAMRMWRDNRKKE (144)
NET73725.1            SsS   (  9) LREKVFQICANLYENNE-KINRDIVREKLGG-GSFTQLSPLVSEWKEQNNQK ( 11)
OBQ42241.1            AfW   (  9) LKDRIFEICENLYKKSE-KVSREKVRSVLGG-GSFSTIGPMIKEWKEQKKVQ (133)
WP_155664073.1        Af    (  6) LKDKIWSIADQLSANGI-NPTNKAVLAELGS-GSYSSISPILKEWREKKNTF (303)
WP_129545793.1        As    ( 10) LKSRIWNIADKLSSEGV-NPTNKVVLNKLGS-GSYSSIAPILKEWREKKHAI (303)
ABK99502.1            PpD2  (  3) -YDKIFEAADKLDFQGK-AVTLASVREVLGG-GSYSTITPALKEWKVRKGAQ (155)
WP_163931603.1        PsS   (  7) -YEQVKQAAEELAAKGE-NPTVDKVRDALGG-GSKSTIAPMLKQWRHQHEQP (272)
WP_126156966.1        AsM   (  8) TYEKVFEIADNLLADGR-RPTQQMVRNELGS-GSLTTINKALNDWWQGLGKR (234)
OUS33959.1            Os    (  6) TSDRVQRAADELLAKGQ-RPTQQAVRNLIGS-GSITTINHALNLWWASLSQR (209)
WP_088952675.1        Vs    ( 33) -LEDVLTAADEIVAKGQ-KPTIERVRQHLGG-GSPNTVSPMLDVWFERLPQR ( 33)
WP_099070060.1        Nl    (104) -REKVFAAADKLFAQGE-IVTKEKVRKEVKG-GSHSTISKYFREWKEQEDNE ( 96)
WP_126349185.1        Er    (  7) IIRSIQDAANALISSGNINPTNAQVCAHLEG-VNISLISPVMREFRARRRIQ (173)

2n5g_KorA             Ec    ( 18) VGQQTIEIARGVLVDGK---PQATFATSLG--LTRGAVSQAVHRVWAAFEDK ( 36)
```

Species abbreviations: Ah, *Aeromonas hydrophila*; Ea, *Enterobacter aerogenes*; Ec, *Escherichia coli*; Ob, *Oceanospirillaceae bacterium*; Hs, *Halomonas sp.*; Kp, *Klebsiella pneumoniae*; Vp, *Vibrio parahaemolyticus*; Mp, *Marinobacter pelagius*; HsM, *Halomonas sp. Marseille-P2426*; Mc, *Moraxella caprae*; bM, *bacterium M00.F.Ca.ET.230.01.1.1*; Mb1, *Moraxellaceae bacterium 17A*; Se, *Salmonella enterica*; Se, *Salmonella enterica*; Ph, *Pseudomonas helleri*; CPc, *Candidatus Paraburkholderia calva*; Xv, *Xanthomonas vasicola pv. vasculorum NCPPB 1381*; C, *Caballeronia*; HsT, *Hydrogenophaga sp. T4*; Kpn, *Klebsiella pneumoniae*; Tn, *Thiothrix nivea*; TsU, *Thauera sp. UPWRP*; Rf, *Rhodoferax ferrireducens*; Xp, *Xanthomonas phaseoli*; Xc, *Xanthomonas citri*; LsD, *Limnohabitans sp. DM1*; Vpa, *Vibrio parahaemolyticus*; Nh, *Nitrosococcus halophilus*; PpA1, *Paenibacillus popilliae ATCC 14706*; PsB, *Pseudochrobactrum sp. B5*; PsD, *Paenibacillus sp. DCT19*; Rm, *Ralstonia mannitolilytica*; Tr, *Thiocapsa roseopersicina*; Pgb, *Patescibacteria group bacterium*; Ea, *Endozoicomonas ascidiicola*; HsQ, *Halomonas sp. QHL1*; CsD, *Crenothrix sp. D3*; MbN, *Methylococcaceae bacterium NSP1-2*; CbB, *Cellvibrionaceae bacterium Bs12*; SsS, *Sphaerospermopsis sp. SIO1G2*; AfW, *Aphanizomenon flos-aquae WA102*; Af, *Aliivibrio fischeri*; As, *Aliivibrio salmonicida*; PpD2, *Pelobacter propionicus DSM 2379*; PsS, *Paraferrimonas sp. SM1919*; AsM, *Amphritea sp. MCCC 1K03512*; Os, *'Osedax' symbiont bacterium Rs2_46_30_T18*; Vs, *Variovorax sp. HW608*; Nl, *Nostoc linckia*; Er, *Enterobacter roggenkampii*; .

Fig S1. A multiple sequence-to-structure alignment of representative KfrA homologs and the HTH domain of KorA from RK2 of IncP. Sequences are tagged according to GenBank accession or PDB code. Residue conservation is denoted using the following scheme: uncharged, highlighted in yellow; polar, highlighted in grey. Numbers of excluded residues are given in parentheses. The predicted secondary alpha-helical structure is shown above the alignment block (H).

Figure S2

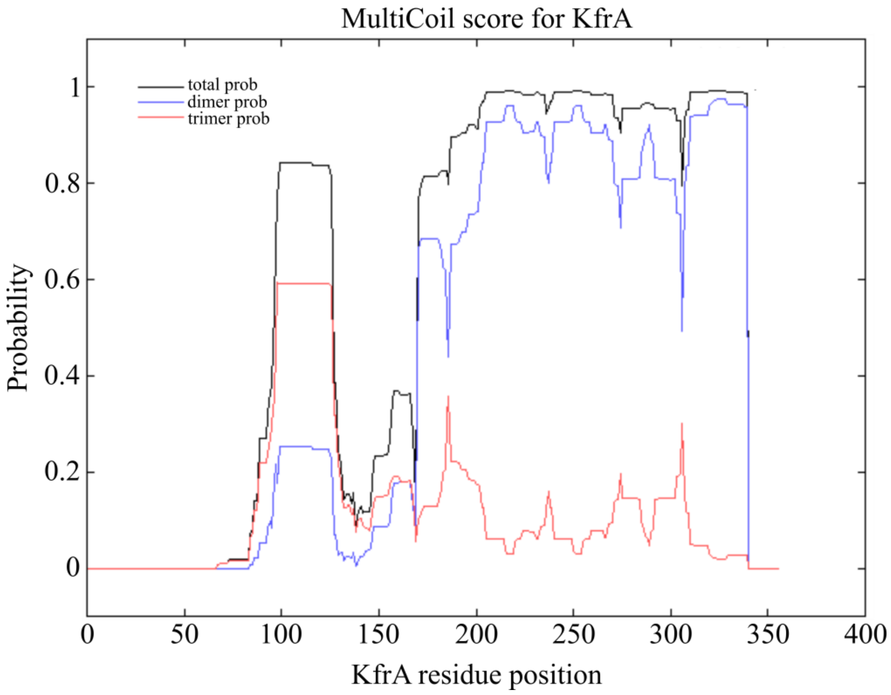

Fig S2. Location of dimeric and trimeric coiled-coil regions in KfrA$_{RA3}$ amino acid sequence predicted with Multicoil [1].

Figure S3

```
                                                  <---------- HTH ---------->
ABD64835.1      RA3       Ah    MTMIKPEMKEKIVRAAESLRSEGVAKPTNEQVRERMGGGSLSHISPVMREWRASQEQSDIVVIELPGELKAGFDRVAAELWQVASKLAAADIEAVKAHAA
AGH89219.1      pMBUI3    ub    MTIIKPEMKEKIVRAAESLRSEGVVKPTNEQVRERMGGGSLSHISPVMREWRASQDQSEIAVIELPGELKAGFDRVAAELWQVASKLAAADIEAVKAHAA
AGH89344.1      pMBUI7    ub    MTMIKPEMKEKIVRAAESLRSEGVVKPTNEQVRERMGGGSLSHISPVMREWRASQDQSEIAVIELPGELKAGFDRVAAELWQVASKLAAADIEAVKAHAA
WP_166464876.1            Ah    MTMIKPEMKEKIVRAAESLRSEGVAKPTNEQVRERMGGGSLSHISPVMREWRASQEQSDIVVIELPGELKAGFDRVAAELWQVASKLAAADIEAVKAHAA
EAZ9531692.1              Se    MTMIKPEMKEKIVRAAESLRAEGVAKPTNEQVRERMGGGSLSHISPVMREWRASQEQSDIVVIELPGELKAGFDRVAAELWQVASKLAAADIEAVKAHAA
MJV49432.1                Se    MTMIKPEMKEKIVRAAESLRAEGVAKPTNEQVRERMGGGSLSHISPVMREWRASQEQSDIVVIELPGELKAGFDRVAAELWQVASKLAAADIEAVKAHAA
WP_101150985.1            Ah    MTMIKPEMKEKIVRAAESLRSEGVAKPTNEQVRERMGGGSLSHISPVMREWRASQEQSDIVVIELPGELKAGFDRVAAELWQVASKLAAADIEAVKAHAA
WP_034284114.1            As    ----------------------------------------------------------------------------------------------------

ABD64835.1      RA3       Ah    EHVAMADQERDEALDEVARLESELDRCRVAVSEKEAETRAALSEKITIEQKAIGLASEVERLTQELAVCRQSIESFTSDN
AGH89219.1      pMBUI3    ub    EHVAMADQERDEALDEVARLEGELDRCRVAVSDKEAETRAALSEKITIEQKAIGLASEVERLTQELAVCRQSIEVFTSDS
AGH89344.1      pMBUI7    ub    EHVAMADQERDEALDEVARLEGELDRCRVAVSDKEAETRAALSEKITIEQKAIGLASEVERLTQELAVCRQSIEVFTSDS
WP_166464876.1            Ah    EHVAMADQERDEALDEVARLESELDRCRVAVSEKEAETRAALSEKITIEQKAIGLASEVERLTQELAVCRQSIESFTSDN
EAZ9531692.1              Se    EHVAMADQERDEALDEVARLESELDRCRVAVSEKEAETRAALSEKITIEQKAIGLASEVERLTQELAVCRQSIESLTSDS
MJV49432.1                Se    EHVAMADQERDEALDEVARLESELDRCRVAVSEKEAETRAALSEKITIEQKAIGLASEVERLTQELAVCRQSIESLTSDS
WP_101150985.1            Ah    EHVAMADQERDEALDEVARLESELDRCRVAVSEKEAETRAALSEKITIEQKAIGLASEVERLTQELAACRQSIESFTSDN
WP_034284114.1            As    --------------------------------------------------------------------------MFTSDS

                                <------------- RPT --------------> <------------- RPT --------------> <------------- RPT -------------->
ABD64835.1      RA3       Ah    ATLTANLKAANQEIDKLAKANQDNQGSIEALKEERATLTANLKAANQEIDKLAKANQDNQGSIEALKEERATLTANLKAANQEIDKLAKANQDNQGSIEALKEER
AGH89219.1      pMBUI3    ub    ATLTANLKAANQEIDKLTKANQDNQGSIEALKEERATLTANLKAANQEIDKLAKANQDNQGSIEALKEERATLTANLKAANQEIDKLAKANQDNQGSIEALKEER
AGH89344.1      pMBUI7    ub    ATLTANLKAANQEIDKLTKANQNNQGSIEALKEERATLTANLKAANQEIDKLAKANQDNQGSIEALKEERATLTAN------------------------------
WP_166464876.1            Ah    ATLTANLKAANQEIDKLAKANQDNQGSIEALKEERATLTANLKAANQEIDKLAKANQDNQGSIEALKEERATLTANLKAANQEIDKLAKANQDNQGSIEALKEER
EAZ9531692.1              Se    ATLTANLKAANQEIDKLAKANQDNQGSIEALKEERATLTANLKAANQEIDKLAKANQDNQGSIEALKEERATLTANLKAANQEIDK-------------------
MJV49432.1                Se    ATLTANLKAANQEIDKLAKANQDNQGSIEALKEERATLTANLKAANQEIDKLTKANQDNQGSIEALKEERATLTASLKAANQEIGKLTKANQDNQGSIEALKEER
WP_101150985.1            Ah    ATLTANLKAANQEIDKL----------------------------------------------------------------------------------------
WP_034284114.1            As    ATLTANLKAANQEIDKLTKANQDNQGSIEALKEERATLTASLKAANQEIDKLTKANQDNQGSIEALKEERATLTASLKAANQEIDKLTKANQDNQGSIEALKEER

                                <------------- RPT --------------> <------------- RPT --------------> <------------- RPT -------------->
ABD64835.1      RA3       Ah    ATLTASLKAANQEIGKLAKANQDNQGSIEALKEERATLTASLKAANQEIDKL-----------------------------------------------------
AGH89219.1      pMBUI3    ub    ATLTASLKAANQEIDKLAKANQDNQGSIEALKEERATLTASLKVANQEIDKL-----------------------------------------------------
AGH89344.1      pMBUI7    ub    ---------------------------------------------------------------------------------------------------------
WP_166464876.1            Ah    ATLTANLKAANQEIDKLAKANQDNQGSIEALKEERATLTANLKAANQEIDKLAKANQDNQGSIEALKEERATLTANLKAANQEIDKLAKANQDNQGSIEALKEER
EAZ9531692.1              Se    --LTANLKAANQGIDKLTKANQDNQGSIEALKEERATLTANLKAANQEIDKLTKANQDNQGSIEALKEERATLTASLKAANQEIGKLTKANQDNQGSIEALKEER
MJV49432.1                Se    ATLTASLKAANQEIEKL----------------------------------------------------------------------------------------
WP_101150985.1            Ah    ---------------------------------------------------------------------------------------------------------
WP_034284114.1            As    ATLTASLKAANQEIDKLTKANQDNQGSIEALKEERATLTASLKAANQEIDKLTKANQDNQGSIEALKEERATLTASLKAANQEIDKLTKANQDNQGSIEALKEER

                                <------------- RPT --------------> <------------- RPT --------------> <------------- RPT -------------->
ABD64835.1      RA3       Ah    ---------------------------------------------------------------------------------------------------------
AGH89219.1      pMBUI3    ub    ---------------------------------------------------------------------------------------------------------
AGH89344.1      pMBUI7    ub    ---------------------------------------------------------------------------------------------------------
WP_166464876.1            Ah    ATLTANLKAANQEIDKLAKANQDNQGSIEALKEERATLTASLKAANQEIDKL-----------------------------------------------------
EAZ9531692.1              Se    ATLTASLKAANQEIEKL----------------------------------------------------------------------------------------
MJV49432.1                Se    ---------------------------------------------------------------------------------------------------------
WP_101150985.1            Ah    ---------------------------------------------------------------------------------------------------------
WP_034284114.1            As    ATLTASLKAANQEIDKLTKANQDNQGSIEALKEERATLTASLKAANQEIDKLTKANQDNQGSIEALKEERATLTASLKAANQEIDKLTKANQDNQGSIEALKEER

                                <------------- RPT --------------> <------------- RPT --------------> <------------- RPT -------------->
ABD64835.1      RA3       Ah    ---------------------------------------------------------------------------------------------------------
AGH89219.1      pMBUI3    ub    ---------------------------------------------------------------------------------------------------------
AGH89344.1      pMBUI7    ub    ---------------------------------------------------------------------------------------------------------
WP_166464876.1            Ah    ---------------------------------------------------------------------------------------------------------
EAZ9531692.1              Se    ---------------------------------------------------------------------------------------------------------
MJV49432.1                Se    ---------------------------------------------------------------------------------------------------------
WP_101150985.1            Ah    ---------------------------------------------------------------------------------------------------------
WP_034284114.1            As    ATLTASLKAANQEIDKLTKANQDNQGSIEALKEERATLTASLKAANQEIDKLTKANQDNQGSIEALKEERATLTASLKAANQEIDKLTKANQDNQGSIEALKEER

                                <------------- RPT --------------> <------------- RPT --------------> <------------- RPT -------------->
ABD64835.1      RA3       Ah    ---------------------------------------------------------------------------------------------------------
AGH89219.1      pMBUI3    ub    ---------------------------------------------------------------------------------------------------------
AGH89344.1      pMBUI7    ub    ---------------------------------------------------------------------------------------------------------
WP_166464876.1            Ah    ---------------------------------------------------------------------------------------------------------
EAZ9531692.1              Se    ---------------------------------------------------------------------------------------------------------
MJV49432.1                Se    ---------------------------------------------------------------------------------------------------------
WP_101150985.1            Ah    ---------------------------------------------------------------------------------------------------------
WP_034284114.1            As    ATLTASLKAANQEIDKLTKANQDNQGSIEALKEERATLTASLKAANQEIDKLTKANQDNQGSIEALKEERATLTASLKAANQEIDKLAKANQDNQGSIEALKEER

                                <------------- RPT -------------->
ABD64835.1      RA3       Ah    -----------------------------------TKPTAKRQQKKDAADTKE [355]
AGH89219.1      pMBUI3    ub    -----------------------------------TKPAAKRQQKKGAIDPKE [355]
AGH89344.1      pMBUI7    ub    ----------------------------------------------------- [256]
WP_166464876.1            Ah    -----------------------------------TKPAAKRQQKKDAADTKE [460]
EAZ9531692.1              Se    -----------------------------------SKPAAKRQQKKDAADIKE [404]
MJV49432.1                Se    -----------------------------------SKPAAKRQQKKDAADTKE [320]
WP_101150985.1            Ah    -----------------------------------TKPAAKRQQKKDAADTKE [215]
WP_034284114.1            As    ATLTASLKVANQEIDKL------------------TKPVAKRQQKKGAIDPKE [566]
```

B

```
                                                         <---------- HTH ---------->
WP_010890107.1  R751      Ea    ( 4)KEQIFAVADELDAA-GQNPTLANVRKQLGSGSFTTISEAMNEWRARKASQAAPIRE(283 α-helical tail)  [342]
BAQ95621.1      pPOP15.9  Pp    ( 8)KEQIWAAAEKLLQEG-KSPTLAAVRGVVGGGSYTTISEAMSEFRAVQEKSDAPIKE(263 α-helical tail)  [326]
NLK11225.1                Seq   (31)QQRIINAANQLVNEGLENPTNLEVRNKMGGGSLSHISPIMRKWREDRKEQVSVALE(131 α-helical tail)  [218]
WP_153956214.1            Cn    (10)RDDVWQAADSLLKAG-QRPTIERIRLHLGRGSPNTVSPHLDAWFAALGGRIQDPQG(256 α-helical tail)  [321]
AAQ97883.1      pEL60     Eam   ( 7)RARIEAAAEALVSEGVNSPTNEQVRQKLGGGSLSHISPVMREWRRRLREQTDTPAL(148 α-helical tail)  [211]
```

Species abbreviations: ub, *uncultured bacterium*; Ah, *Aeromonas hydrophila*; Se, *Salmonella enterica*; As, *Aeromonas salmonicida*; Ea, *Enterobacter aerogenes*; Pp, *Paenibacillus popilliae*; Seq, *Staphylococcus equorum*; Cn, *Cupriavidus necator*; Eam, *Erwinia amylovora*.

Fig. S3. Multiple sequence alignments for KfrA homologs discussed in the manuscript. Sequences are tagged according to GenBank accession identifier, followed by plasmid name, if applicable. **(A)** Representative full-length KfrA proteins sharing iterative alpha-helical tail. HTH domains and sequence

repeats are marked above the corresponding alignment blocks. Predicted alpha-helices are denoted in red. Sequence lengths are provided in square brackets. **(B)** HTH domains of chosen KfrA homologs with alpha helical tails. Numbers of excluded residues are given in parentheses. Total protein lengths are shown in square brackets.

1. Construction of truncated *kfrA* alleles

RA3 was used as a template in all PCRs to amplify *kfrA* fragments with primers providing start and stop codons, when required, as well as additional restriction sites to enable joining the fragments in different combinations. *kfrA* and its deletion alleles were first constructed in the cloning vectors pBGS18 or pUC18 and after sequence verification re-cloned into expression plasmids pGBT30 and pET28mod.

$kfrA_{1\text{-}53}$, $kfrA_{1\text{-}177}$, $kfrA_{54\text{-}177}$ and $kfrA_{178\text{-}355}$ were amplified with the use of primer pairs 30/36, 32/33, 30/33 and 34/35, respectively.

Additionally, $kfrA_{1\text{-}53}$ without a stop codon was amplified with the use of a primer pair 30/31 in order to construct $kfrA_{\Delta 54\text{-}177}$ and WT *kfrA* alleles. $kfrA_{\Delta 54\text{-}177}$ was assembled by joining $kfrA_{1\text{-}53}$ without a stop codon with a $kfrA_{178\text{-}355}$ using the Ecl136II and EheI restriction sites. $kfrA_{54\text{-}355}$ was assembled by joining $kfrA_{54\text{-}177}$ with $kfrA_{178\text{-}355}$ using SnaBI and Ecl136II restriction sites. The WT *kfrA* allele was assembled by joining $kfrA_{1\text{-}53}$ without a stop codon with $kfrA_{54\text{-}355}$ using Ecl136II and EheI restriction sites.

kfrA deletion alleles were re-cloned as EcoRI-SalI fragments into pGBT30 and pET28mod. These derivatives of pGBT30 and pET28mod are listed in Table 1.

2. Cloning into the bacterial two-hybrid (BACTH) vectors

$kfrA_{1\text{-}53}$, $kfrA_{54\text{-}177}$, $kfrA_{178\text{-}355}$, $kfrA_{1\text{-}177}$, $kfrA_{\Delta 54\text{-}177}$, $kfrA_{54\text{-}355}$ and *kfrA* obtained as described above were into cloned into the bacterial adenylate cyclase two-hybrid (BACTH) vectors pLKB4 and/or pLKB2 to create translational fusions of the respective proteins via their the N-terminus with the CyaA T18 and T25 domains.

To create hybrid proteins linked to the CyaA T25 and T18 domains via the C-terminus, first the appropriate *kfrA* alleles without stop codons were constructed:

- $kfrA_{1\text{-}53}$, $kfrA_{1\text{-}177}$, $kfrA_{54\text{-}177}$ and $kfrA_{178\text{-}355}$ were amplified with the use of primer pairs 30/89, 30/90, 32/90 and 34/91, respectively.
- $kfrA_{\Delta 54\text{-}177}$ was assembled by joining $kfrA_{1\text{-}53}$ without a stop codon with a $kfrA_{178\text{-}355}$ without a stop codon using the Ecl136II and EheI restriction sites.
- $kfrA_{54\text{-}355}$ was assembled by joining $kfrA_{54\text{-}177}$ with $kfrA_{178\text{-}355}$ without a stop codon using SnaBI and Ecl136II restriction sites.
- WT *kfrA* allele was assembled by joining $kfrA_{1\text{-}53}$ without a stop codon with $kfrA_{54\text{-}355}$ using Ecl136II and SnaBI restriction sites.

Next, *kfrA* and the deletion alleles were re-cloned into pKGB4 and/or pKGB5.

kfrC without a stop codon was re-cloned from pAKB2.55 into pKGB4 and pKGB5 to create translational fusions with the CyaA T25 and T18 domains via the C-terminus. Then, the stop codon was

introduced downstream of the *kfrC* coding sequence in pAKB2.55 on annealed oligonucleotides 95 and 96, and the obtained *kfrC* allele was re-cloned into pLKB4 and pLKB2 to create translational fusions via the N-terminus with the CyaA T18 and T25 domains.

All pLKB2, pLKB4, pKGB4 and pKBG5 derivatives are listed in Table 1.

3. Construction of translational fusions of His_6 with KfrA via N-terminus and FLAG with KfrC via C-terminus.

kfrC without a stop codon was amplified with primers 100/101 and joined with *kfrA* in pKAB21 to create translational fusions of encoded KfrA and KfrC with His_6- and FLAG-tags, respectively. Finally, *his_6-kfrA kfrC-flag* was re-cloned into pET28a derivative (pKAB28) as an EcoRI-SalI fragment.

4. Construction of translational fusions of His_6 with KfrC via C-terminus

pET28a(+) was modified to enable cloning a gene of interest as an EcoRI-HindIII fragment to create translational fusion with *his6* tag via the C-terminus of the encoded protein. To that end, annealed oligonucleotides 99 and 100 were inserted between BamHI and NcoI restriction sites of pET28a(+) to give pESB15. The HindIII recognition sequence was inserted downstream of *kfrC* in pAKB2.55 on annealed oligonucleotides 97 and 98. Finally, *kfrC* was re-cloned as an EcoRI-HindIII fragment into pESB15 to give pESB15.90.

5. Detailed description of co-immunoprecipitation of KfrA with KfrC

The formaldehyde-treated cells from 100 ml were harvested by centrifugation for 10 minutes at 5000xg at 4°C, washed twice with 10 ml of ice-cold PBS buffer (15 mM KCl, 150 mM NaCl and 10 mM NaPi, pH 7.4) and resuspended in 0.5 ml of the lysis buffer [10 mM Tris-HCl (pH 8.0), 20% (w/v) sucrose] with lysozyme (1 mg/ml). After 30 minutes of incubation on ice, 200 µl of IP buffer [1.5 M Tris-HCl (pH 7.0), 0.3 M NaCl, 0.2% (v/v) Triton X-100], protease inhibitor cocktail (Sigma) and PMSF (1 mM) were added and the samples were incubated on ice for 10 minutes and then 5 minutes more at room temperature. Cells were disrupted by sonication and the cell extracts were centrifuged twice at 15000xg for 20 minutes at 4°C. Cleared extracts (20-120 µl) were added to the mixture of immunoprecipitation buffer and lysis buffer in proportion 2:5 supplemented with 5 µg of anti-FLAG antibodies (Invitrogen), 1 U of viscolase (A&A Biotechnology) and 10 mM $MgCl_2$, in a final volume of 500 µl. Samples were incubated overnight at 4°C with gentle agitation. Subsequently, 50 µl of A-sepharose (GE Healthcare) were added and after 2 hours of incubation at room temperature on rotating shaker suspensions were washed 8 times with the lysis buffer. The proteins content was analyzed by western blotting.

References

1. Wolf E, Kim PS, Berger B. 1997. MultiCoil: a program for predicting two- and three-stranded coiled coils. Protein Sci 6:1179–1189.
